# Pyrroloquinoline quinone drives ATP synthesis in vitro and in vivo and provides retinal ganglion cell neuroprotection

**DOI:** 10.1186/s40478-023-01642-6

**Published:** 2023-09-08

**Authors:** Alessio Canovai, James R. Tribble, Melissa Jöe, Daniela Y. Westerlund, Rosario Amato, Ian A. Trounce, Massimo Dal Monte, Pete A. Williams

**Affiliations:** 1 Division of Eye and Vision, Department of Clinical Neuroscience, St. Erik Eye Hospital, Karolinska Institutet, Stockholm, Sweden; 2 Department of Biology, University of Pisa, Pisa, Italy; 3 Department of Surgery, Centre for Eye Research Australia, Royal Victorian Eye and Ear Hospital, Ophthalmology, University of Melbourne, Melbourne, VIC Australia

**Keywords:** Pyrroloquinoline quinone (PQQ), Mitochondria, Metabolism, Metabolomics, ATP, Retinal ganglion cell, Retina, Optic nerve, Neuroprotection

## Abstract

Retinal ganglion cells are highly metabolically active requiring strictly regulated metabolism and functional mitochondria to keep ATP levels in physiological range. Imbalances in metabolism and mitochondrial mechanisms can be sufficient to induce a depletion of ATP, thus altering retinal ganglion cell viability and increasing cell susceptibility to death under stress. Altered metabolism and mitochondrial abnormalities have been demonstrated early in many optic neuropathies, including glaucoma, autosomal dominant optic atrophy, and Leber hereditary optic neuropathy. Pyrroloquinoline quinone (PQQ) is a quinone cofactor and is reported to have numerous effects on cellular and mitochondrial metabolism. However, the reported effects are highly context-dependent, indicating the need to study the mechanism of PQQ in specific systems. We investigated whether PQQ had a neuroprotective effect under different retinal ganglion cell stresses and assessed the effect of PQQ on metabolic and mitochondrial processes in cortical neuron and retinal ganglion cell specific contexts. We demonstrated that PQQ is neuroprotective in two models of retinal ganglion cell degeneration. We identified an increased ATP content in healthy retinal ganglion cell-related contexts both in in vitro and in vivo models. Although PQQ administration resulted in a moderate effect on mitochondrial biogenesis and content, a metabolic variation in non-diseased retinal ganglion cell-related tissues was identified after PQQ treatment. These results suggest the potential of PQQ as a novel neuroprotectant against retinal ganglion cell death.

**Supplementary Information:**

The online version contains supplementary material available at 10.1186/s40478-023-01642-6.

## Introduction

Retinal ganglion cells (RGCs) are the output neurons of the retina, the axons of which form the optic nerve, connecting the eye to the brain. RGCs are highly metabolically and physiologically active cells, requiring a constant supply of ATP to ensure proper function. A finely regulated metabolism and perfectly balanced mitochondrial function are fundamental to maintain ATP at a physiological level. Imbalances in these mechanisms can be detrimental to RGC viability by depleting ATP, rendering RGCs susceptible to damage [1]. Altered metabolism and mitochondrial abnormalities have been reported to occur early in the pathogenesis of many optic neuropathies, including glaucoma, autosomal dominant optic atrophy (ADOA), and Leber hereditary optic neuropathy (LHON), where progressive RGC dysfunction and degeneration are typical hallmarks of disease [2–8]. Reducing bioenergetic insufficiency by buffering metabolic stress, positively regulating mitochondrial mechanisms, and restoring ATP levels have all been demonstrated to be neuroprotective to RGCs [7, 9]. A treatment that can prevent metabolic dysfunction and arrest RGC degeneration is highly sought after. There is strong potential for bioenergetic compounds which could be supplemented in diet, adjuvant to existing medication to increase RGC resilience, and with better side effect profiles.

Pyrroloquinoline quinone (PQQ) is a quinone cofactor first described in bacterial dehydrogenases [10]. It has been considered as a ‘new vitamin’ given its nutritional importance on mammalian growth, reproduction, and development demonstrated by a wide range of abnormalities when PQQ is absent in the diet [11, 12]. PQQ is not synthetized de novo in mammals, but is instead present in several foods, such as parsley, green pepper, spinach, kiwi, and soybeans, thus giving the possibility to consume PQQ through dietary supplementation [13]. PQQ administration has a good safety profile, with a median LD50 of 0.5–2.0 g/kg in rats, and no signs of toxic effects when treated long term via oral gavage [14]. This suggests a strong potential for PQQ as a compound to support retinal ganglion cell health.

Several studies have reported an effect of PQQ on metabolism and mitochondrial mechanisms, acting as an enzyme cofactor [15], regulating nicotinamide adenine dinucleotide (NAD) content [16], increasing oxidative phosphorylation (OXPHOS) and ATP production [17], and altering mitochondrial dynamics and content through the regulation of pathways particularly involved in mitochondrial biogenesis [18, 19]. However, such effects are highly context-dependent and heterogeneous, dictating the necessity to study, in depth, the mechanism of PQQ in specific systems. Although there is some evidence suggesting a putative neuroprotection of PQQ in models of acute damage in the central nervous system (CNS) [20–23], the role of PQQ in metabolism and mitochondrial content in a neuronal context has not been extensively investigated.

In this study, we explored neuroprotection exerted by PQQ in models of RGC degeneration where bioenergetic capacity is compromised. Given the reported ability of PQQ to provide bioenergetic support in other systems, we hypothesized that PQQ could provide neuroprotection by bolstering bioenergetic support. Supporting this hypothesis, we demonstrate that PQQ is neuroprotective in two models of RGC degeneration. In addition, we identify that PQQ administration leads to increased neuronal ATP content in RGC-related tissues both in in vitro and in vivo models. Although we identified a mild effect exerted by PQQ on mitochondrial biogenesis and content, we report a metabolic variation in non-diseased RGC-related tissues. Taken together, these results suggest a potential role of PQQ as a novel neuroprotective compound to improve RGC resilience.

## Materials and methods

### Animal strain and husbandry

All breeding and experimental procedures were managed in accordance with the Association for Research for Vision and Ophthalmology Statement for the Use of Animals in Ophthalmic and Research. Individual study protocols were accepted by Stockholm’s Committee for Ethical Animal Research (10389-2018). Animals were housed in a regulated environment, (12 h light/12 h dark cycle) and fed with food and water ad libitum. C57BL/6J (B6J) and MitoV (strain information detailed below) mouse strains were bred and used at 12–20 weeks of age. PQQ disodium salt (Mitsubishi Gas Chemical Company Inc., Tokyo, Japan) was dissolved in DMSO and diluted in Hank’s balanced salt solution (without CaCl_2_, MgCl_2_ and phenol red) (HBSS; Gibco). Mice were treated with either vehicle (HBSS) or 20 mg/kg PQQ i.p. either for a single injection (short-term) or chronically every 48 h for 2 weeks (long-term) as further indicated. In a subset of experiments, PQQ was dissolved in drinking water at the concentration of 0.2 mg/mL in order to obtain a daily administration of 20 mg/kg considering an individual mean drinking volume of 3 mL/day. Water was protected from light as PQQ is light sensitive.

### Retina axotomy explant model

A retinal axotomy model was performed as previously reported [7]. Briefly, B6J mice (n = 13) were euthanized by cervical dislocation, retinas immediately dissected in ice-cold HBSS and flat mounted on inserts for cell culture (Millicell 0.4 µm pore; Merck) with ganglion cell layer (GCL) up. Retinas were cultured (37 °C, 5% CO_2_) in media composed of Neurobasal-A supplemented with 2 mM L-glutamate (GlutaMAX, Gibco), 2% B27, 1% N2, and 1% penicillin/streptomycin (Gibco) in 6-well culture plates. After 2 days, half of the media volume was replaced. For PQQ treated retinas, PQQ was dissolved in the culture media to a concentration of 50 or 100 μM. Retinas were removed from culture to be further processed after 3 days ex vivo (DEV). For controls (0 DEV), eyes were enucleated and retinas directly fixed and processed for immunofluorescent labelling.

### Intravitreal rotenone model

Rotenone induced retinal degeneration model was performed following established protocols [7]. B6J mice (n = 20) underwent anesthesia by an intraperitoneal injection of mixed ketamine (37.5 mg/kg) and medetomidine hydrochloride (1.25 mg/kg). Two µl of 10 mM rotenone diluted in DMSO (all Sigma-Aldrich) or DMSO only (control) was bilaterally injected into the vitreous using a 33G tri-beveled needle on a 10 µl glass syringe (WPI). For PQQ treated retinas, PQQ (20 mg/kg) i.p. was administered 24 h prior to and immediately after rotenone injection. Mice were euthanized by cervical dislocation 24 h after rotenone injection and eyes were immediately isolated for further analyses.

### Analysis of RGC death and degeneration

Loss of RGCs, loss of nuclei, and nuclear shrinkage in retinal explants (n = 5 retinas from 0 DEV, 9 from 3 DEV, 4 from 3 DEV + 50 μM, 6 from 3 DEV + 100 μM) and rotenone injected retinas (n = 9 DMSO, 9 DMSO + PQQ, 10 rotenone and 9 rotenone + PQQ) were assessed by immunofluorescence through RBPMS and DAPI labelling of flat mounts. Retinal explants at 3 DEV were fixed in 3.7% PFA for 30 min, detached from the cell culture insets and transferred on slides. Eyes from the animals injected with rotenone were fixed in PFA at the same concentration for 1 h immediately after enucleation. Retinas were then dissected in ice-cold HBSS and transferred onto slides. Tissues were isolated using a hydrophobic barrier pen (VWR) and permeabilized with 0.5% Triton X-100 (VWR) in 1 M PBS for 1 h. After blocking in 2% bovine serum albumin (BSA, Fisher Scientific) in 1 M PBS for 1 h, retinas were immunolabelled with rabbit polyclonal anti-RBPMS (NBP2-20112, Novusbio, 1:500) primary antibody at 4 °C overnight. Thereafter, retinas were rinsed by 5 washes in 1 M PBS for 5 min each before labelling with Alexa Fluor 568 conjugated goat anti-rabbit secondary antibody (A11011, Invitrogen, 1:500) for 4 h at room temperature. Tissue was then washed as before and counterstained with DAPI (1 μg/mL in 1 M PBS) for 10 min. After being rinsed once in PBS, tissue was mounted with Fluoromount-G and glass coverslips (Invitrogen). Nail-varnish was used to seal the slides which were kept at 4 °C until further imaging. The acquisition of the images was performed on a Leica DMi8 microscope with a CoolLED pE-300 white LED-based light source and a Leica DFC7000 T fluorescence color camera (all Leica). Six images per retina (40× magnification) were acquired equidistantly at 0, 2, 4, 6, 8, and 10 o’clock from a superior to inferior line through the optic nerve head at an eccentricity of around 1000 μm. Images were cropped to 100 μm × 100 μm squares and the cell counter plugin in Fiji was used to count RBPMS + cells and DAPI nuclei (only round nuclei were considered, thus discarding vascular endothelium). The mean of cell counts per retina was measured across the 6 images and expressed as a density per 0.01 mm^2^. For the measurement of nuclear diameter, 30 nuclei from RBPMS-positive cells (or in as many surviving cells in the image) per cropped image were quantified using the line tool, providing an average diameter. The mean diameter from each cropped image was then averaged across the 6 images to obtain a final average diameter per retina.

### Luminometry-based ATP and NAD assays

ATP or NAD content was analyzed in vitro and in vivo following a similar protocol. In order to assess how quickly PQQ could be used by cells in vitro to increase ATP and NAD content, ATP and NAD assays were run on treated brain cortex first to make a dose–response curve and then on retina, optic nerve and superior colliculus exposed to a single concentration. A total of 4 hemispheres were harvested from the whole cortex of B6J mice. Each hemisphere was transferred to 800 µl of dispase (5000 U; Corning) and incubated on a Thermomixer C heating block (Eppendorf) at 37 °C, 350 rpm for 30 min before being dissociated by gentle trituration. Cell concentration was calculated by cell counting on a C-Chip hemocytometer (NanoEntek) and each cell suspension was diluted to 2 million cells/mL. Seven aliquots of each cell suspension for each sample were incubated with different concentrations of PQQ (0.1, 0.5, 1, 5, 10, 50 μM) for 2 h at 37 °C, 5% CO_2_. Cells maintained in HBSS for the same time were used as controls. Samples were then homogenized for 15 s at 30,000 min^−1^ (VDI 12, VWR) and ATP or NAD content was measured using a luminometry-based assay (CellTiter-Glo® Luminescent Cell Viability Assay for ATP, NAD/NADH Glo-™ for NAD; Promega). Reagents of the kit were prepared according to the manufacturer’s instructions. Equal volumes (50 μl) of sample (reaching a concentration of 100,000 cells/well) and working reagent were combined in a 96-well plate (Nunc™ F96 MicroWell™ White Polystyrene plate, Thermo Fisher Scientific). Luminescence was measured using a Tecan Infinite 200 at approximately 10 min for ATP and 1 h for NAD from initial mixing according to the manufacturer’s instructions. To exclude any possible interaction between PQQ and ATP working reagent, the assay was run on PQQ solutions diluted in HBSS without cell lysates as a positive control, demonstrating no change in luminescence with increasing concentrations of PQQ (Additional file 2: Fig. 1). To measure the in vitro ATP content on treated retina, optic nerve and superior colliculus, 5 B6J mice were euthanized by cervical dislocation, whole eyes enucleated, and retinas dissected in HBSS. The brain was removed, and the superior colliculus was isolated. Optic nerves were cut at 3 mm from the end proximal to the eye, left and right segments were collected in two different samples and stored at 4 °C in HBSS until further processing. Retinas from left and right eyes were pooled to form a single sample. Retinas and superior colliculi were dissociated in 500 and 800 µl of dispase respectively and processed as described above. Cells from dissociated retinas and superior colliculi, as well as isolated optic nerve segments, were incubated with 50 μM PQQ for the same time and in the same conditions as above. The bioluminescent assay was then conducted as previously described. The assessment of ATP and NAD content in vivo was performed on B6J mice (n = 36) treated short-term with an injection of either vehicle or 20 mg/kg PQQ. A cohort of animals (n = 12) was administered with either water or PQQ dissolved in drinking water at the same dose. Animals were euthanized at 24, 48, 72 h after the injection and 24 h after the water administration. Brains, retinas, optic nerves, and superior colliculi were collected and processed as described above. A direct homogenization after the cell dilution was performed and the luminescent assay was run with the same procedure previously indicated.

### Trypan Blue assay

Cell viability was assessed by Trypan Blue staining. Cells from brain cortex were obtained and diluted as above. Samples were incubated with 50 μM PQQ for 2 h, stained with 0.4% Trypan Blue dye (Thermo Fisher Scientific) and counted on a hemocytometer (NanoEntek).

### JC-1 staining

To measure the short-term effects of PQQ on mitochondrial membrane potential (∆Ψ), JC-1 staining on treated brain cortical cells (dose–response), or retina, optic nerve and superior colliculus (single dose) was performed according to previous protocols with some variations [24]. Briefly, a total of 4 hemispheres from whole cortexes of B6J mice were harvested and brain cortical cells were obtained as described above. Cell suspensions were diluted at a concentration of 1 million cells/mL, then aliquots from each sample were incubated with solutions of PQQ at different concentrations (0.5, 5, 50 μM) for 2 h. At the last 30 min of incubation, JC-1 (Thermo Fisher Scientific, dissolved in DMSO) was added at a final concentration of 2 μM and cells were kept in the dye until the end of PQQ incubation. Cells were washed from the dye, resuspended in HBSS and 50 μl of sample was loaded on a 96-well plate (Nunc™ F96 MicroWell™ White Polystyrene plate, Thermo Fisher Scientific) in an alternate manner to avoid interference between adjacent wells. Fluorescence at 535 nm (green; monomer) and 590 nm (red; aggregate) was measured using a Tecan Infinite 200 and ΔΨ levels were expressed as the ratio of red to green fluorescence. To measure ∆Ψ levels in treated retina, optic nerve and superior colliculus, B6J mice (n = 8 for retinas, 5 for the other tissues) were euthanized and the tissues collected as described in the previous section. Cells from retinas and superior colliculi were diluted at a concentration of 1 million cells/mL and incubated with 50 μM PQQ for 2 h. JC-1 was administered at the same concentration and times used for cortical cells. Optic nerves were incubated with PQQ for 1 h, homogenized in PQQ at 8000 min^−1^ speed (VDI 12, VWR) and incubated with 2 μM JC-1 for 30 min at 37 °C for a total of 2 h of incubation in PQQ. Fluorescence was measured as previously described.

### Mitochondrial isolation and individual mitochondrial complex activity assays

A total of 6 hemispheres from whole brain cortexes of B6J mice were isolated and dissociated as previously detailed. Cells were then diluted at a concentration of 4 million cells/mL and incubated with 50 μM PQQ for 2 h. Thereafter, mitochondria were extracted using the Thermo Scientific™ Mitochondria Isolation Kit for Cultured Cells (Thermo Fisher Scientific) according to the manufacturer’s instructions. Isolated mitochondria were then suspended in 80 μL of mitochondrial isolation buffer (220 mM mannitol, 70 mM sucrose, 10 mM Tris–HCL, 1 mM EDTA, pH = 7.2) and protein content quantified by Pierce™ Detergent Compatible Bradford Assay Kit (Thermo Fisher Scientific). Samples were then sonicated with 3 pulses of 3 s each at ~ 4 W, aliquoted and kept at − 80 °C until further processing. Assays to evaluate the activity of individual mitochondrial complexes were performed according to the protocols described in the supplementary materials from [25], with some adjustments for plates and plate reader. All the assays were performed in a 96 well plate (Sarstedt) and the readings run using a Tecan Infinite 200. Briefly, for the Complex (C) II assay, 20–80 μg/mL of mitochondrial proteins were incubated in 20 mM succinate, 2 μg/mL rotenone, 2 μg/mL antimycin A, 2 mM KCN, 50 μM DCPIP (all Sigma-Aldrich) in potassium buffer for 10 min and then the reduction of DCPIP was measured after the addition of 10 mM DB (Sigma-Aldrich) at 595 nm for 3 min. To assess the activity of CIII, 20 μg/mL of mitochondrial proteins were incubated in 50 μM decylubiquinol, 2 mM KCN, 50 μM cytochrome C (Cyt C) (all Sigma-Aldrich) in sucrose/Tris buffer and the reduction of Cyt C was monitored at 550 nm for 3 min. To account for CIII-independent Cyt C reduction, samples were run both without or with 2 μg/mL antimycin A. Regarding the CIV assay, 10 μg/mL of mitochondrial proteins were mixed with 20 μM ferrocytochrome c (FeCyt C) in degassed potassium buffer, and the oxidation of FeCyt C was measured at 550 nm for 3 min. For the citrate synthase (CS) assay, 20 μg/mL of mitochondrial proteins were incubated with 100 μM DTNB, 300 μM acetyl-CoA and 500 μM oxaloacetate (Sigma-Aldrich) in Tris buffer, and the reduction of DTNB was checked at 415 nm for 3 min. The enzyme activity was calculated by the formula: enzyme activity (nmol min^−1^ mg^−1^) = (ΔAbsorbance/min × 1000)/[(extinction coefficient × volume of sample used in mL) × (sample protein concentration in mg mL^−1^)] [26]. The ΔAbsorbance/min was derived from the slope of the linear phase of the reaction. The specific activity of CIII was calculated by subtracting the activity without antimycin A minus the one in presence of the inhibitor. The activities of CII, III and IV were normalized to the CS.

### Quantitative real-time PCR

B6J treated with either vehicle or 20 mg/kg PQQ i.p. either short-term (n = 7 mice for 24 h, 8 for the other time points) or long-term (n = 16 mice) were euthanized by cervical dislocation. For the short-term treatment, retinas were collected 24, 48 and 72 h after the injection, whereas for the long-term administration retinas and optic nerves were harvested 15 days after the starting point. Eyes were enucleated and retinas dissected in ice-cold HBSS. A second operator isolated the optic nerves from the brain and cut around the optic chiasm. A single retina comprised of a sample, whereas left and right optic nerve segments were pooled together to make a unique sample. All the samples were then snap frozen in dry ice and stored at − 80 °C until further processing. A total RNA extraction was performed homogenizing retinas and optic nerves in 350 µl buffer RLT (Qiagen) with 1% β-mercaptoethanol (Fisher Scientific) and using a QIAshredder kit (Qiagen) according to the manufacturer’s instructions. A column-based kit (RNeasy Mini Kits, Qiagen) was used to extract RNA according to the manufacturer’s instructions. Isolated RNA was suspended in nuclease-free water and RNA concentration was quantified using a NanoDrop™ One (Thermo Fisher Scientific). cDNA was generated starting from 1 μg of input RNA through an iScript™ cDNA Synthesis Kit and a MyIQ thermocycler (both Bio-Rad) and stored at − 20 °C. Quantitative real-time PCR was performed at the CFX96 Touch Real-Time PCR Detection System thermocycler (Bio-Rad) using SsoAdvanced Universal SYBR Green Supermix (Bio-Rad), cDNA (15 ng for retina; 3 ng for optic nerve) and the appropriate DNA templates (Prime PCR Assay, *mus musculus*, Bio-Rad): *mt-Co2*, *Rsp18*, *Pgc-1α*, *Tfam*, *Ndufb8*, *Sdhb*, *Uqcrc2*, *mt-Co1*, *Atp5a1*. The protocol used comprised of a 3 min activation and denaturation step at 95 °C, followed by an amplification stage composed by 50 cycles of a 15 s denaturation at 95 °C and 1 min annealing, and plate read at 60 °C. Data was exported and opened in the software CFX manager (Bio-Rad) to visualize the amplification and melting curves. The expression levels were calculated by the ΔΔCt method. The mtRNA/nuRNA ratio was calculated using *mt-Co2* and *Rsp18* as the reference mitochondrial and nuclear gene, respectively, as described in [27]. *Rsp18* was used as housekeeping gene when the expression of the other genes was calculated.

### Western blot

B6J mice (n = 16) treated with intraperitoneal injections of either vehicle or 20 mg/kg PQQ long-term were euthanized by cervical dislocation after 15 days from the starting point. Eyes were enucleated and retinas dissected in ice-cold HBSS. A second operator isolated the optic nerves from the brain and segments were obtained by cutting right before the optic chiasm. Left and right retinas or optic nerves from the same animal were pooled to make a single sample. All the samples were then snap frozen in dry ice and stored at − 80 °C until further processing. Samples were lysed in RIPA buffer (Santa Cruz Biotechnology) supplemented with phosphatase and proteinase inhibitor cocktails (Roche Applied Science). Protein content was measured by Micro BCA protein assay (Thermo Fisher Scientific). Equal micrograms of proteins per sample (10 µg for retinas; 20 µg for optic nerves) were separated by SDS-PAGE (4–20%; Bio-Rad) and gels were transblotted onto nitrocellulose membranes (Bio-Rad). Membranes were blocked with either 4% BSA (Sigma-Aldrich) or 5% skim milk in TBS/Tween for 1 h at room temperature and incubated overnight at 4 °C with mouse monoclonal total OXPHOS rodent WB antibody cocktail (ab110413, Abcam, 1:250, recognizing 5 different targets: NDUFB8, SDHB, UQCRC2, mt-CO1, ATP5a) or mouse monoclonal anti-β-actin antibody (A2228, Sigma-Aldrich, 1:2500). Afterward, membranes were incubated for 2 h at room temperature with the solution of HRP-conjugated rabbit polyclonal anti-mouse antibody (A9044, Sigma-Aldrich, 1:5000). Rat heart mitochondrial extract provided by the manufacturer (ab110341, Abcam) was diluted at 1:200 and used as a positive control. Clarity western chemiluminescence substrate (Bio-Rad) was used to develop blots, and images were acquired by the ChemiDoc™ Imaging System (Bio-Rad). Protein levels of the target bands were expressed by normalizing the optical density (OD) of the target calculated by Image Lab 6.0 software (Bio-Rad) to the corresponding OD of β-actin as loading control.

### Mitochondrial morphological analyses

To image and analyze mitochondrial morphology, retinal and optic nerve cryo-sections were cut from MitoV mice (n = 8) treated for 2 weeks in vivo with either vehicle or 20 mg/kg PQQ i.p. The MitoV mouse strain is on a B6J background and expresses YFP under a rat neuron-specific *Eno2* promoter. YFP is localized to mitochondria through a *Cox8a* gene-targeting signal fused to the YFP N-terminus. This line was selected due to its specificity in inner retinal expression (defined MitoV for visual tissue) [7]. The strain has been further characterized in previous reports [7]. Mice were euthanized after 15 days from the first injection and eyes enucleated. The brain was isolated with the attached optic nerves. Tissues were fixed in 3.7% PFA for 24 h and cryo-protected by immersion in 30% sucrose solution. Eyes were directly frozen in optimal cutting temperature medium (Sakura) on dry ice, whereas optic nerves were separated from the brain and then included in the same medium. Blocks were maintained at − 80 °C until use. Eyes and optic nerves were cryo-sectioned in 20 μm-thick coronal and longitudinal sections respectively using a cryostat (Cryostar NX70, Thermo Scientific). All the sections were stored at − 20 °C until further processing. Cryo-sections were then air dried for 15 min and rehydrated in 1 M PBS for 5 min before following the protocol. Tissues were isolated using a hydrophobic barrier pen (VWR), permeabilized with 0.1% Triton X-100 (VWR) in 1 M PBS for 1 h and blocked in 2% BSA (Fisher Scientific) in 1 M PBS for 1 h. Sections were then immunolabelled with chicken polyclonal anti-GFP (ab13970, Abcam, 1:500) and rabbit polyclonal anti-TOMM20 (ab78547, Abcam, 1:500) primary antibodies at 4 °C overnight. Anti-GFP primary antibody was used to limit loss of signal from potential bleaching of YFP. Thereafter, sections were rinsed with 5 × 5 min washes in 1 M PBS and stained with Alexa Fluor 488 conjugated goat anti-chicken (A11039, Invitrogen, 1:500) and Alexa Fluor 568 conjugated goat anti-rabbit (A11011, Invitrogen, 1:500) secondary antibodies for 4 h at room temperature. Tissue was washed as before and counterstained with DAPI (1 μg/mL in 1 M PBS) for 10 min. After being rinsed once in PBS, tissue was mounted using Fluoromount-G and glass coverslips (Invitrogen). Nail-varnish was used to seal the slides. Images were acquired using confocal imaging on a Zeiss LSM-980 Airy (63×, 1.5 × optical zoom, image size 89.8 × 89.8 μm, 0.07 µm pixel size, z-stacks with 0.23 μm optimal interval). Images from retinal sections were acquired from central retina at ~ 500 μm lateral to the optic nerve head. Images from optic nerve sections were acquired from areas around the optic chiasm used for reference. Alexa Fluor 488 conjugated and Alexa Fluor 568 conjugated secondary antibodies targeting anti-GFP and anti-TOMM20 primary antibodies respectively were imaged. DAPI channel was also imaged for reference purposes in retinal samples. Retinal images encompassed nerve fiber layer (NFL), GCL and inner plexiform layer (IPL). Mitochondrial particles were reconstructed in 3D using Imaris software (version 9.3.1). NFL/GCL and IPL were cropped for the analyses and reconstructed separately with different settings. Volume reconstructions were performed using the surface tool and volumes under 125 voxels were filtered and discarded from subsequent analysis to reduce noise. Volume, surface area, sphericity (including prolate and oblate dimensions) of each mitochondrial particle were calculated by the software and plotted either individually or as an average per retina or optic nerve. For volume and surface area, an average of both mean and sum of each parameter was calculated. Mean volume, volume sum, mean surface area and surface area sum in each retina were normalized to the number of either GFP or TOMM20 positive cells and then to the volume crop in NFL/GCL, or to only volume crop in IPL. The same parameters were normalized to the volume crop in the optic nerve images.

### Metabolomics

B6J mice (n = 10 animals) treated with a single i.p. injection of either vehicle or 20 mg/kg PQQ were euthanized by cervical dislocation after 24 h from the treatment. Eyes were enucleated and retinas were immediately dissected in ice cold HBSS, wiped dry, weighed and frozen on dry ice. A second investigator isolated the optic nerves immediately after death. Optic nerves were cut at 3 mm from the end proximal to the eye and frozen as for retinas. Each sample comprised a single retina or an isolated optic nerve segment. Tissue was stored at − 80 °C and shipped kept in dry ice to the Swedish Metabolomics Centre for sample processing. 200 μl of extraction buffer (80:20 v/v MeOH:H_2_O) including internal standards were added to the tubes together with 1 tungsten bead. Tissues were shaken at 30 Hz for 3 min in a mixer mill and samples were then centrifuged at 4 °C, 18620 g for 10 min. Afterwards, 200 μl of the supernatant was transferred to micro vials and evaporated to dryness in a speed-vac concentrator. Samples were stored at − 80 °C and small aliquots of the remaining supernatants were pooled and used to create quality control (QC) samples. Prior to the analysis, samples were re-suspended in 10 + 10 µl methanol and elution solvent A. The samples were analyzed in batches according to a randomized run order. Each batch of samples was first analyzed in positive mode. After all samples within a batch had been analyzed, the instrument was switched to negative mode and a second injection of each sample was performed. The chromatographic separation was performed on an Agilent 1290 Infinity UHPLC-system (Agilent Technologies, Waldbronn, Germany). 2 μl of each sample were injected onto an Atlantis Premier BEH-Z-HILIC VanGuard FIT (1.7 µm, 2.1 × 50 mm) column (Waters Corporation, Milford, MA, USA) held at 40 °C. The HILIC gradient elution solvents were (A) 10 mM ammonium formate, 5 µM Medronic acid in H_2_O, pH 9 and (B) 90:10 Acetonitrile: [10 mM ammonium formate in H_2_O], pH 9. Chromatographic separation was achieved using a linear gradient (flow rate of 0.4 mL/ min): min 0 = 90% B, min 6 = 80% B, min 9.5 = 20% B, min 11 = 90% B. The flow rate was then increased to 0.7 mL/min for 2 min, held at this rate for 0.5 min, and further reduced to 0.4 mL/min for 0.5 min before the next injection. Compounds were detected with an Agilent 6546 Q-TOF mass spectrometer equipped with a jet stream electrospray ion source operating in positive or negative ion mode. MSMS analysis was run on the QC samples for identification purposes. All data pre-processing was performed using the Agilent MassHunter Profinder version B.10.0 SP1 (Agilent Technologies Inc., Santa Clara, CA, USA). Sixty-three (retina) or seventy-three (optic nerves) low molecular weight metabolites that could be certified with standards were detected. The quantification of the metabolites was calculated as area under the curve of the mass spectrometry peak and normalized first to an internal standard for negative and positive runs, then for the weight of the tissue for retinal samples. Data were analyzed and graphs were made using MetaboAnalyst [version 5.0; 28, 29] and R. All data were subject to Pareto scaling [30]. Hierarchical clustering (HC) (Spearman, Average) was used to create the dendrograms. Correlation heatmaps were created using Spearman rank correlation. Principal component analysis (PCA) was performed in R (4.1.0) using the *factoextra* package. Comparisons between groups were analyzed by two-sample t-tests with an adjusted *p* value (false discovery rate, FDR), using a cutoff of 0.05 considered significant. Quantitative pathway analysis was performed using the *Mus musculus* KEGG library in MetaboAnalyst and a background metabolome of all detected metabolites.

### Statistical analysis

Graph Pad Prism 8.0.2 software and R were used for the statistical analyses. A Shapiro Wilk test was used to test the normality of the data. A Student’s t-test or one-way ANOVA (followed by Tukey’s multiple comparison post hoc test) were applied as appropriate to analyze normally distributed data. A Mann–Whitney test was used to analyze non-normally distributed data. For individual mitochondrial particles morphology, a linear mixed effects model through the lme4 package in R was applied when multiple observations come from the same retina or optic nerve, in order to reduce p value inflation and intra-class correlation [31–33]. Differences with *p* < 0.05 were considered significant. For the box plots, the median is represented by the center hinge with upper and lower hinges indicating the first and third quartiles, whereas whiskers denote 1.5 times the interquartile range. All the graphs were made in R or using MetaboAnalyst 5.0 for dendrograms and correlation heatmaps.

## Results

### PQQ is neuroprotective in different models of RGC-related damage

**Fig. 1:**
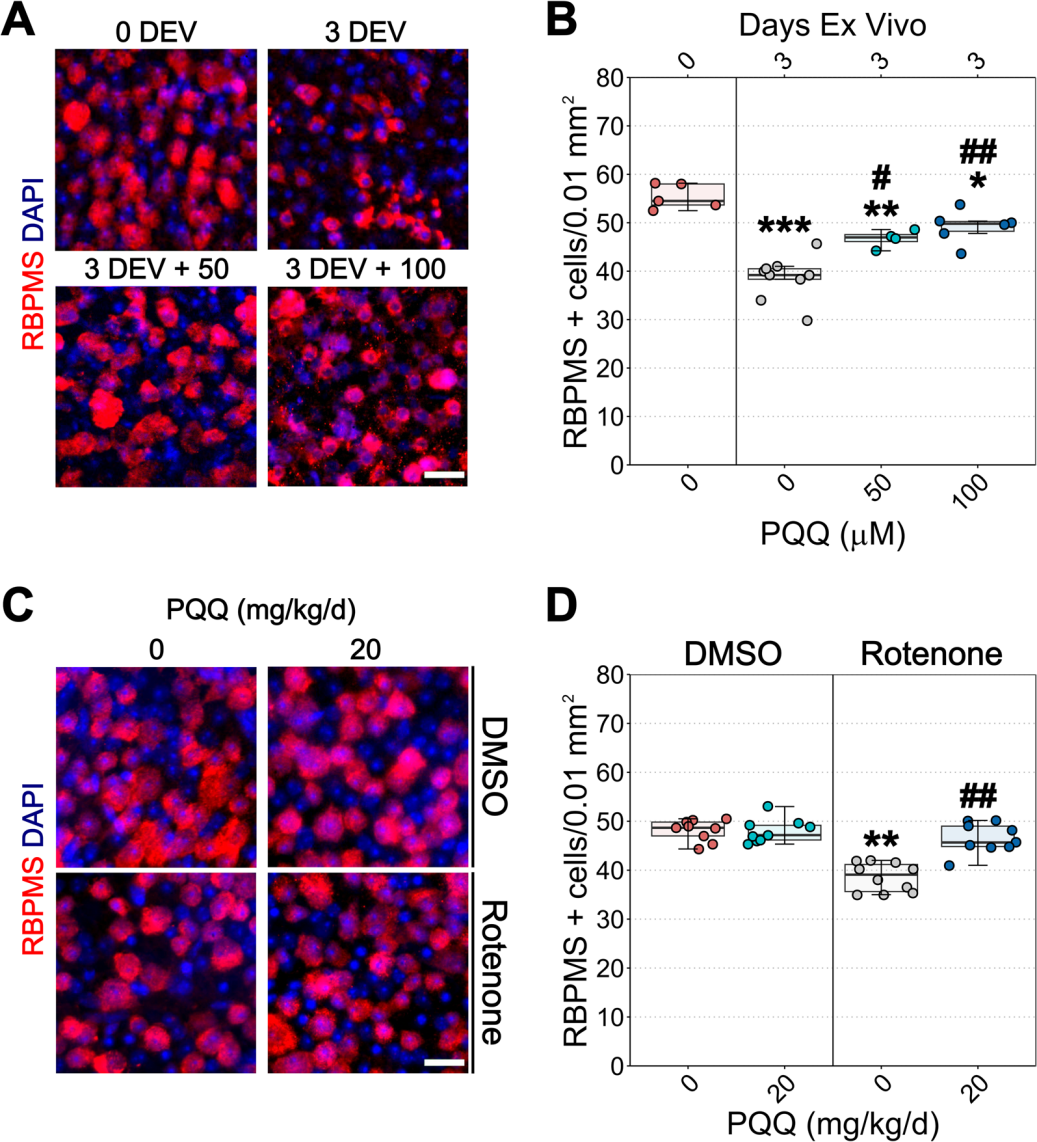
Effects of PQQ administration on RGC survival in ex vivo and in vivo models of RGC stress. **A** Representative images of retinas cultured ex vivo, immunolabeled for RNA-binding protein with multiple splicing (RBPMS, red) and counterstained with DAPI (blue). Retinal explants were cultured in either basic or supplemented media with either 50 or 100 μM PQQ for 3 days ex vivo (DEV). Control retinas (0 DEV) were directly fixed and processed after the dissection. **B** Quantification of RBPMS positive cell density per 0.01 mm^2^. n = 5 (0 DEV), 9 (3 DEV), 4 (3 DEV + 50 μM PQQ), 6 (3 DEV + 100 μM PQQ) retinas. **C** Representative images of retinas from mice injected with either DMSO or rotenone and treated with either vehicle or 20 mg/kg PQQ. Flat mount retinas were immunolabeled for RBPMS (red) and counterstained with DAPI (blue). **D** Quantification of RBPMS positive cell density per 0.01 mm^2^. n = 9 DMSO, 9 DMSO + PQQ, 10 rotenone and 9 rotenone + PQQ retinas. Scale bar = 20 μm. *p < 0.05, **p < 0.01 and ***p < 0.001 versus 0 DEV (explants) or DMSO (rotenone model); ^#^*p* < 0.01 and ^##^*p* < 0.001 versus 3 DEV (explants) or rotenone (rotenone model)

Since RGCs strictly rely on a perfectly controlled metabolism and PQQ has been demonstrated to regulate the cellular bioenergetic balance [15–19], we hypothesized that PQQ could protect RGCs under stress/injury which compromise bioenergetic capacity. We first evaluated the effects of PQQ in an ex vivo model of RGC injury, which reproduces an axon-specific insult by separating the retina from the optic nerve [7, 34]. Significant RGC loss was detected after 3 days ex vivo (DEV), as assessed by counting cells positive for RBPMS (a specific marker of RGCs). Administration of PQQ via the culture media at both 50 and 100 μM provided a significant preservation of RGC density under stress condition (Fig. 1A, B). The administration of PQQ was effective in counteracting the loss of DAPI positive nuclei, however the nuclear shrinkage was only partially prevented at the highest dose (Additional file 3: Fig. 2A, B).

To further explore PQQ neuroprotection, we next tested the ability of PQQ to protect RGC against Complex I inhibition in a model of RGC degeneration induced by rotenone. In this model, retinal cell death is induced by the inhibition of mitochondrial Complex I triggered by an intravitreal injection of rotenone, resulting in a rapid degeneration of retinal neurons [7, 35]. A significant RGC and retinal cell loss, as well as nuclear shrinkage, were identified after 24 h from the rotenone injection (Fig. 1C, D; Additional file 3: Fig. 2C, D). While the administration of PQQ was effective in preventing RGC and retinal cell loss, the nuclear shrinkage was persisting in retinas of PQQ-treated mice although significantly attenuated (Fig. 1C, D; Additional file 3: Fig. 2C, D). These data support a neuroprotective role of PQQ against a range of different stressors, such as RGC axonal damage and severe mitochondrial dysfunction.

### PQQ increases ATP content and alters mitochondrial membrane potential in cortical neurons and RGCs in vitro

**Fig. 2:**
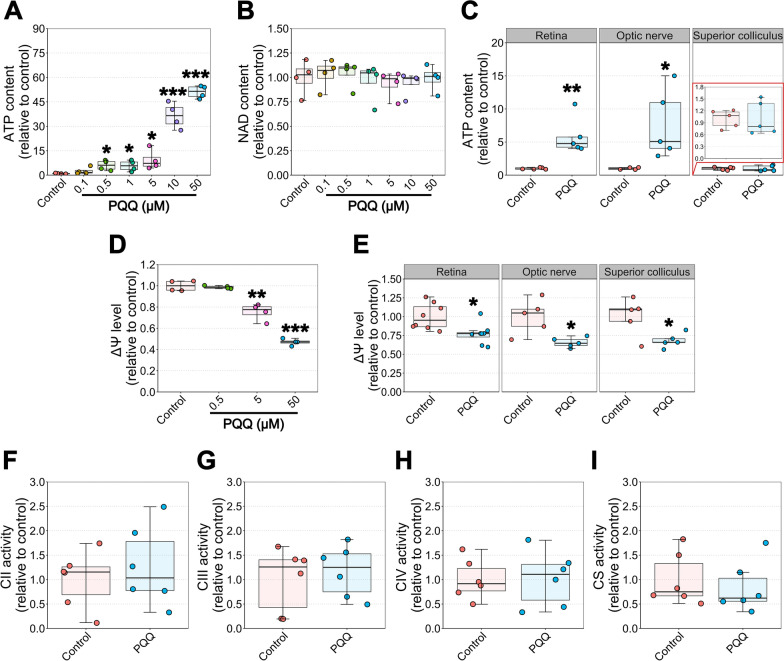
Effects of PQQ administration on ATP, NAD, mitochondrial membrane potential levels and mitochondrial function in vitro. **A** ATP and **B** NAD content in dissociated mouse brain cortical cells incubated with different doses of PQQ (0.1, 0.5, 1, 5, 10, 50 µM) for 2 h. n = 4 different cell suspensions from different hemispheres. **C** ATP content in dissociated retinal and superior colliculus cells and in isolated optic nerves incubated with 50 µM PQQ for 2 h. The red inset shows a zoom of the graph related to the ATP content in superior colliculus. n = 5 retinal replicates made by 2 pooled retinas, 5 optic nerve replicates made of isolated segments cut to 3 mm length, 5 superior colliculus replicates. **D** Mitochondrial membrane potential (ΔΨ) levels in dissociated brain cortical cells incubated with several concentrations of PQQ (0.5, 5, 50 µM) for 2 h. n = 4 different cell suspensions from different hemispheres. **E** ΔΨ levels in dissociated retinal and superior colliculus cells and in isolated optic nerves incubated with 50 µM PQQ for 2 h. n = 8 retinal replicates made by 2 pooled retinas, 5 optic nerve replicates made of isolated segments cut to 3 mm length, 5 superior colliculus replicates. **F**–**I** Mitochondrial Complex (**C**) II (**F**), III (**G**), IV (**H**) and citrate synthase (CS; **I**) activity in dissociated cortical cells incubated with 50 µM PQQ for 2 h. n = 6 different cell suspensions from different hemispheres. *p < 0.05, **p < 0.01 and ***p < 0.001 versus control

As PQQ has been reported to influence metabolism and regulate the cellular bioenergetic balance in several systems [15–19], we investigated the effect of PQQ on neuronal ATP production. We initially assessed if PQQ was rapidly utilized by measuring the ATP content in dissociated brain cortical cells. Cortical cells were incubated with increasing concentrations of PQQ (0.1, 0.5, 1, 5, 10, 50 μM) for 2 h and ATP levels assessed. A significant dose-dependent increase in ATP content was demonstrated in cortical cells from concentrations at 0.5 μM, reaching > 50-fold the untreated control at the highest dose tested (Fig. 2A) without a loss of viability (Additional file 4: Fig. 3). As NAD is a key cofactor involved in metabolism and ATP synthesis, we assessed if the short incubation with PQQ might promote NAD synthesis. However, PQQ effect on generating ATP in vitro appeared not predominantly dependent on NAD synthesis, since NAD content was not significantly influenced following the incubation with PQQ (Fig. 2B). To support these findings in an RGC specific context we next assessed if PQQ at 50 μM (where the highest ATP increase was demonstrated) provided a similar ATP increase in RGC-related tissues. Dissociated retinal cells and optic nerve confirmed the increase in ATP as seen in cortical neurons (Fig. 2C).

To further investigate the mechanisms behind PQQ’s rapid ATP increase in vitro, we assessed mitochondrial membrane potential (ΔΨ) in cortical cells incubated with PQQ for 2 h. The levels of ΔΨ dose-dependently reduced in PQQ-incubated cells, decreasing to 47% of the control at 50 μM (Fig. 2D). The decline in ΔΨ levels was further confirmed in dissociated retina, optic nerve, or superior colliculus incubated at a dose of 50 μM (Fig. 2E), suggesting that, together with a concomitant increase in ATP levels, mitochondrial potential may be dissipated to produce ATP [24]. Since ΔΨ is highly influenced by the activity of mitochondrial complexes and enzymes from tricarboxylic acid (TCA) cycle (such as citrate synthase; CS), we next assessed a direct interaction between PQQ and each individual mitochondrial complex. Mitochondria were isolated from cortical cells incubated with PQQ and spectrophotometric assays to measure the activity of Complexes II–IV (CII, CIII, CIV) and CS were performed. No significant change in individual complexes and citrate synthase activity was detected suggesting that PQQ does not act directly in the mitochondrion itself (Fig. 2F–I). Taken together, these data suggest that cells from CNS and RGC-related tissues can quickly use PQQ and increase their ATP content.

### PQQ modulates ATP and NAD content in visual system tissues in vivo

**Fig. 3:**
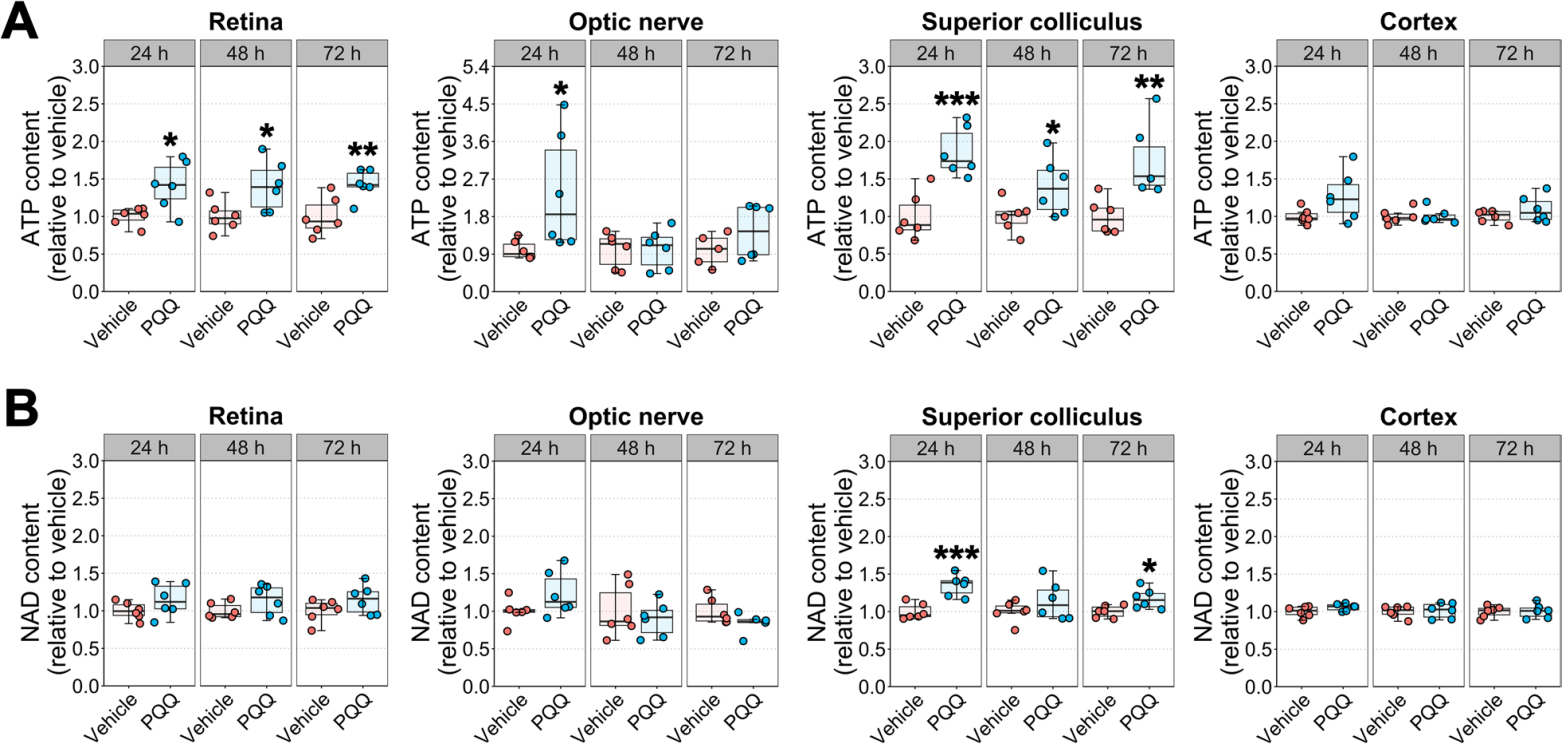
Effects of PQQ administration on ATP and NAD levels in visual system tissues in vivo. **A** ATP and **B** NAD content in retina, optic nerve, superior colliculus, and brain cortex from animals treated with either vehicle or a single injection of 20 mg/kg PQQ assessed after 24, 48 or 72 h from the treatment. n = 6 animals per group for each time point. *p < 0.05, **p < 0.01 and ***p < 0.001 versus vehicle

We next assessed whether the administration of PQQ in vivo leads to similar effects on metabolism. Adult B6J mice were treated with PQQ and the levels of ATP and NAD in visual system tissues were assessed following a single intraperitoneal injection of 20 mg/kg PQQ. A significant increase in ATP levels in retina, optic nerve, and superior colliculus (a major target for RGC axons in the brain) from PQQ-treated animals was identified after 24 h. Higher levels of ATP in PQQ-treated mice were also detected in retinas and superior colliculi after 48 and 72 h (Fig. 3A). PQQ administration significantly increased NAD levels in the superior colliculus after 24 and 72 h from the treatment, whilst having no effect on the other tissues at all the time points analyzed (Fig. 3B). Since we confirmed an increase in ATP in vivo after the treatment with PQQ, we next assessed the efficacy of PQQ when administered in normal drinking water. However, ATP and NAD levels were not elevated by dietary PQQ (except for a significant increase in superior colliculus NAD), suggesting ineffective bioavailability of PQQ orally (Additional file 5: Fig. 4).

### PQQ exerts a mild effect on mitochondrial content and morphology in RGCs

ATP production is strictly dependent on mitochondrial activity and content, and PQQ has been previously reported to regulate mitochondrial biogenesis through the activation of peroxisome proliferator-activated receptor-gamma coactivator alpha (PGC-1α) and the expression of mitochondrial transcription factor A (TFAM) [18, 19]. We initially assessed this short-term transcriptional activation of mitochondrial biogenesis in the whole retina at the same time points where ATP increases were identified in vivo. We performed qPCR to assess the mtRNA:nuRNA ratio, which provides an estimate of the number of mitochondrial genome copies, an early hallmark of mitochondrial biogenesis and an indicator of the number of mitochondria [27]. The expression of *Pgc-1α* and *Tfam* were also assessed. Short-term treatment with PQQ did not effect mtRNA:nuRNA or *Pgc-1α* and *Tfam* mRNA levels, suggesting that a single injection of PQQ is not adequate to induce transcriptional variations in retinal mitochondrial biogenesis (Additional file 6: Fig. 5).

**Fig. 4:**
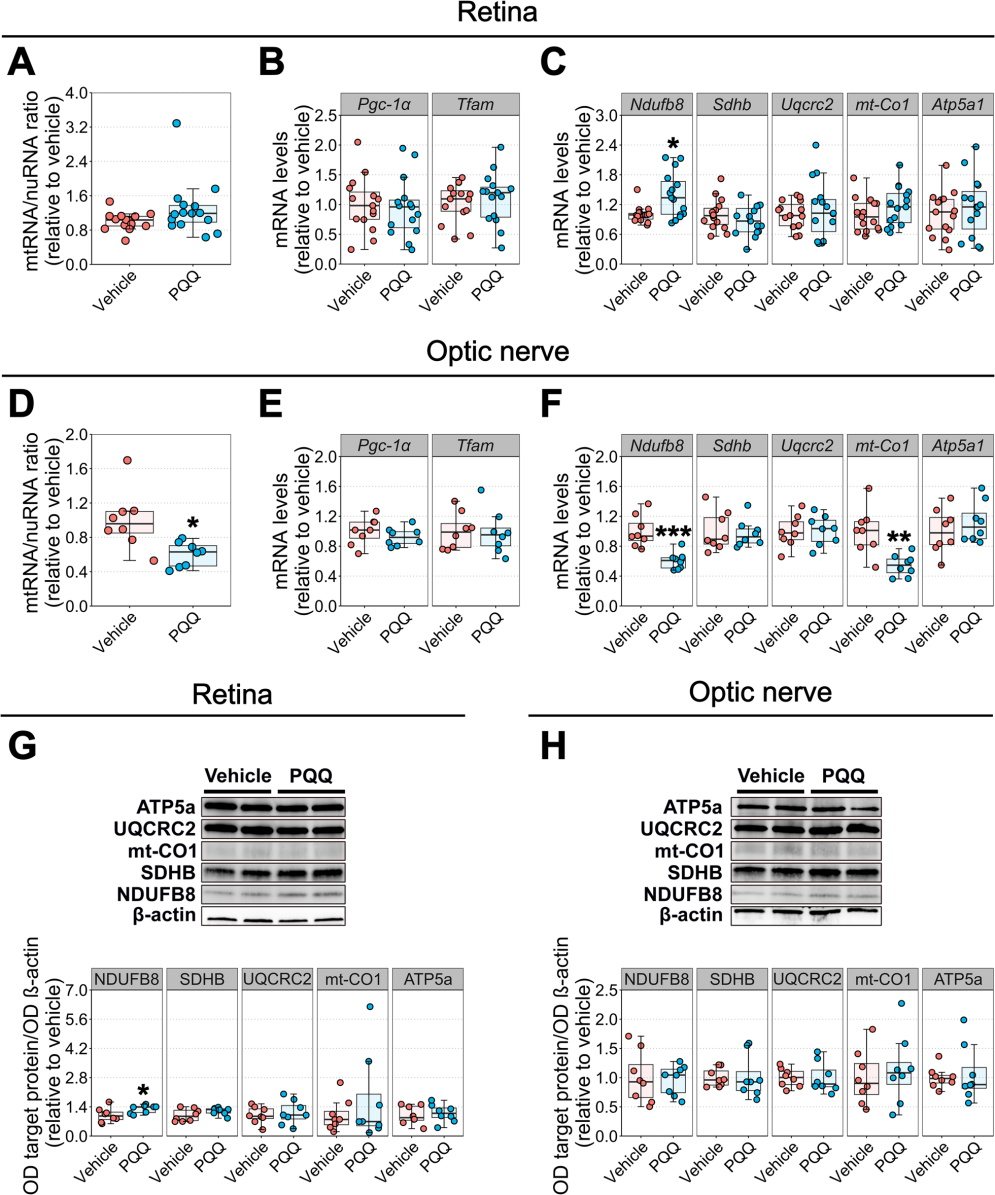
Effects of PQQ administration on molecular regulation of mitochondrial content in vivo. **A** mtRNA/nuRNA ratio in retinal samples from animals treated with either vehicle or 20 mg/kg PQQ long-term, calculated using the expression of *mt-Co2* and *Rsp18* as mitochondrial and nuclear reference gene, respectively. **B**, **C** *Pgc-1α*, *Tfam* (**B**) and *Ndufb8*, *Sdhb*, *Uqcrc2*, *mt-Co1* and *Atp5a1* (**C**) mRNA levels in whole retinas from animals injected long-term with either vehicle or 20 mg/kg PQQ. *Rsp18* was used as housekeeping gene. n = 15 retinas per group. **D** mtRNA/nuRNA ratio in optic nerve samples from animals treated long-term with either vehicle or 20 mg/kg PQQ, calculated using the same genes described in **A** as mitochondrial and nuclear reference genes. **E**, **F** *Pgc-1α*, *Tfam* (**E**) and *Ndufb8*, *Sdhb*, *Uqcrc2*, *mt-Co1* and *Atp5a1* (**F**) mRNA levels in optic nerves from animals treated long-term with either vehicle or 20 mg/kg PQQ. *Rsp18* was used as housekeeping gene. n = 8 optic nerves per group. **G**, **H** Representative blots and densitometric analysis of NDUFB8, SDHB, UQCRC2, mt-CO1 and ATP5a protein levels in retinas (**G**) and optic nerves (**H**) from vehicle and PQQ treated animals. Protein levels were expressed as the optical density (OD) of the target normalized for the respective OD of β-actin used as loading control. n = 8 samples per group. *p < 0.05, **p < 0.01 and ***p < 0.001 versus vehicle

We next questioned whether transcriptional regulation of mitochondria is activated if PQQ was administered chronically. In addition to *Pgc-1α*, *Tfam*, and the mtRNA:nuRNA ratio, we quantified the expression of individual mitochondrial complexes-related genes in retinal samples to further assess an influence on mitochondrial content. mRNA levels of *Ndufb8* (CI), *Sdhb* (CII), *Uqcrc2* (CIII), *mt-Co1* (CIV), *Atp5a1* (CV; ATP synthase) were assessed. Chronic administration of PQQ had no effect on either retinal mtRNA:nuRNA ratio or *Pgc-1α* and *Tfam* mRNA, suggesting that PQQ is not effective in triggering mitochondrial biogenesis transcriptionally short-term or long-term (Fig. 4A, B). However, PQQ-treated retinas displayed a significant increase in *Ndufb8* mRNA levels, without a change in other mitochondrial markers (Fig. 4C). Since whole retinas contain different mixed cell populations, we next assessed if PQQ could induce a similar response in the optic nerve, which is an RGC enriched tissue (RGC axons). A significant decrease in mtRNA:nuRNA ratio, as well as *Ndufb8* and *mt-Co1* mRNA levels, was identified in optic nerves from PQQ-injected mice (Fig. 4D–F). To further confirm these molecular regulations, protein levels were quantified by Western blot to assess if the observed transcriptional changes were strictly correlated with variations in protein levels. A significant increase in NDUFB8 protein levels was demonstrated in retinas from PQQ-treated animals, suggesting that its transcriptional regulation consequently results in its protein translation (Fig. 4G; Additional file 7: Fig. 6A, C). However, no changes in the levels of mitochondrial complexes were identified in the optic nerve after PQQ treatment (Fig. 4H; Additional file 7: Fig. 6B, D).

**Fig. 5:**
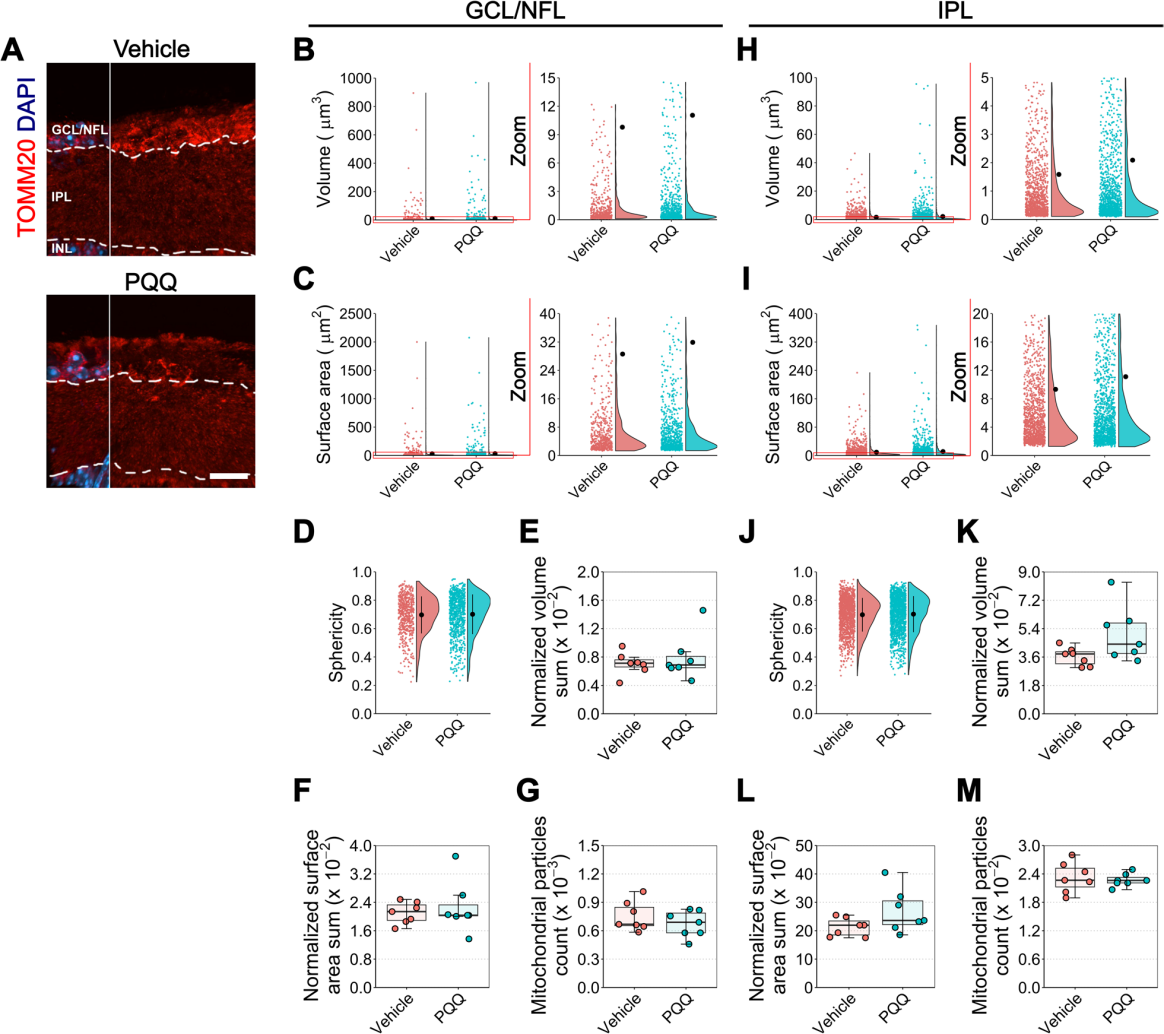
Effects of PQQ administration on retinal gross mitochondrial morphology in vivo. **A** Representative images of retinal cross sections from animals treated long-term with either vehicle or 20 mg/kg PQQ immunolabeled for TOMM20 (red) and counterstained with DAPI (blue). The DAPI was used for reference to crop GCL/NFL from IPL. Dashed lines demark the boundaries between GCL/NFL, IPL and INL on retinal sections. **B**–**D** Violin plots representing individual volume (**B**), surface area (**C**) and sphericity (**D**) of TOMM20-positive mitochondrial particles in GCL/NFL of vehicle or PQQ-treated animals. n = 555 vehicle and 812 PQQ disconnected particles from 7 different retinas per group. **E**–**G** Averaged volume sum (**E**), surface area sum (**F**) and count (**G**) per retina of TOMM20-positive mitochondrial particles in GCL/NFL. n = 7 retinas per group. **H**–**J** Violin plots showing individual TOMM20-positive mitochondrial particles volume (**H**), surface area (**I**) and sphericity (**J**) in IPL of vehicle or PQQ-treated animals. n = 1318 vehicle and 1361 PQQ disconnected particles from 7 different retinas per group. **K**–**M** Averaged volume sum (**K**), surface area sum (**L**) and count (**M**) per retina of TOMM20-positive mitochondrial particles in IPL. n = 7 retinas per group. For individual parameters, individual values of disconnected particles from each retina were analyzed together and a linear mixed effects model was applied to account for the multiple observations that come from the same sample. Scale bar = 20 μm. GCL, ganglion cell layer; INL, inner nuclear layer; IPL, inner plexiform layer; NFL, nerve fiber layer. The red zoom depicts the inset of data points to optimally visualize the data distribution

**Fig. 6:**
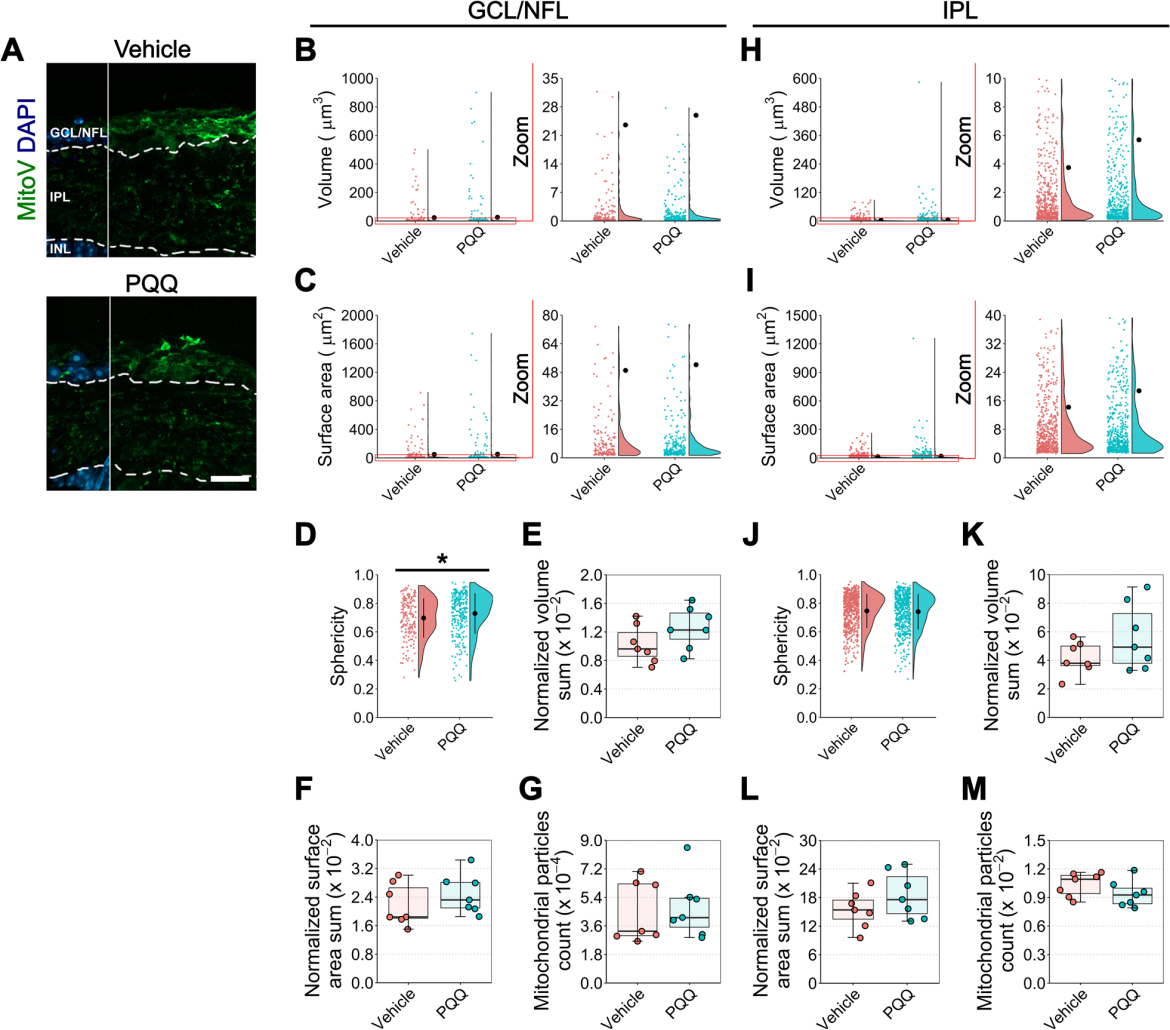
Effects of PQQ administration on RGC-specific retinal mitochondrial morphology in vivo. **A** Representative images of retinal cross sections from MitoV animals treated long-term with either vehicle or 20 mg/kg PQQ immunolabeled for YFP (an antibody anti-GFP was used to limit loss of signal from potential bleaching of YFP; the staining is indicated here as MitoV) (green) and counterstained with DAPI (blue). The DAPI was used for reference to crop GCL/NFL from IPL. Dashed lines demark the boundaries between GCL/NFL, IPL and INL on retinal sections. (B-D) Violin plots depicting individual volume (**B**), surface area (**C**) and sphericity (**D**) of MitoV-positive mitochondrial particles in GCL/NFL of animals treated with either vehicle or PQQ. n = 176 vehicle and 268 PQQ disconnected particles in 7 different retinas per group. **E**–**G** Averaged volume sum (**E**), surface area sum (**F**) and count (**G**) per retina of MitoV-positive mitochondrial particles in GCL/NFL. n = 7 retinas per group. **H**–**J** Violin plots showing individual volume (**H**), surface area (**I**) and sphericity (**J**) of MitoV-positive mitochondrial particles in IPL of vehicle and PQQ-treated animals. n = 600 vehicle and 552 PQQ disconnected particles in 7 different retinas per group. **K**–**M** Averaged volume sum (**K**), surface area sum (**L**) and count (**M**) per retina of MitoV-positive mitochondrial particles in IPL. n = 7 retinas per group. For individual parameters, individual values of disconnected particles from each retina were analyzed together and a linear mixed effects model was applied to account for the multiple observations that come from the same sample. Scale bar = 20 μm. GCL, ganglion cell layer; INL, inner nuclear layer; IPL, inner plexiform layer; NFL, nerve fiber layer. The red zoom depicts the inset of data points to optimally visualize the distribution of the data. *p < 0.05 versus vehicle

**Fig. 7:**
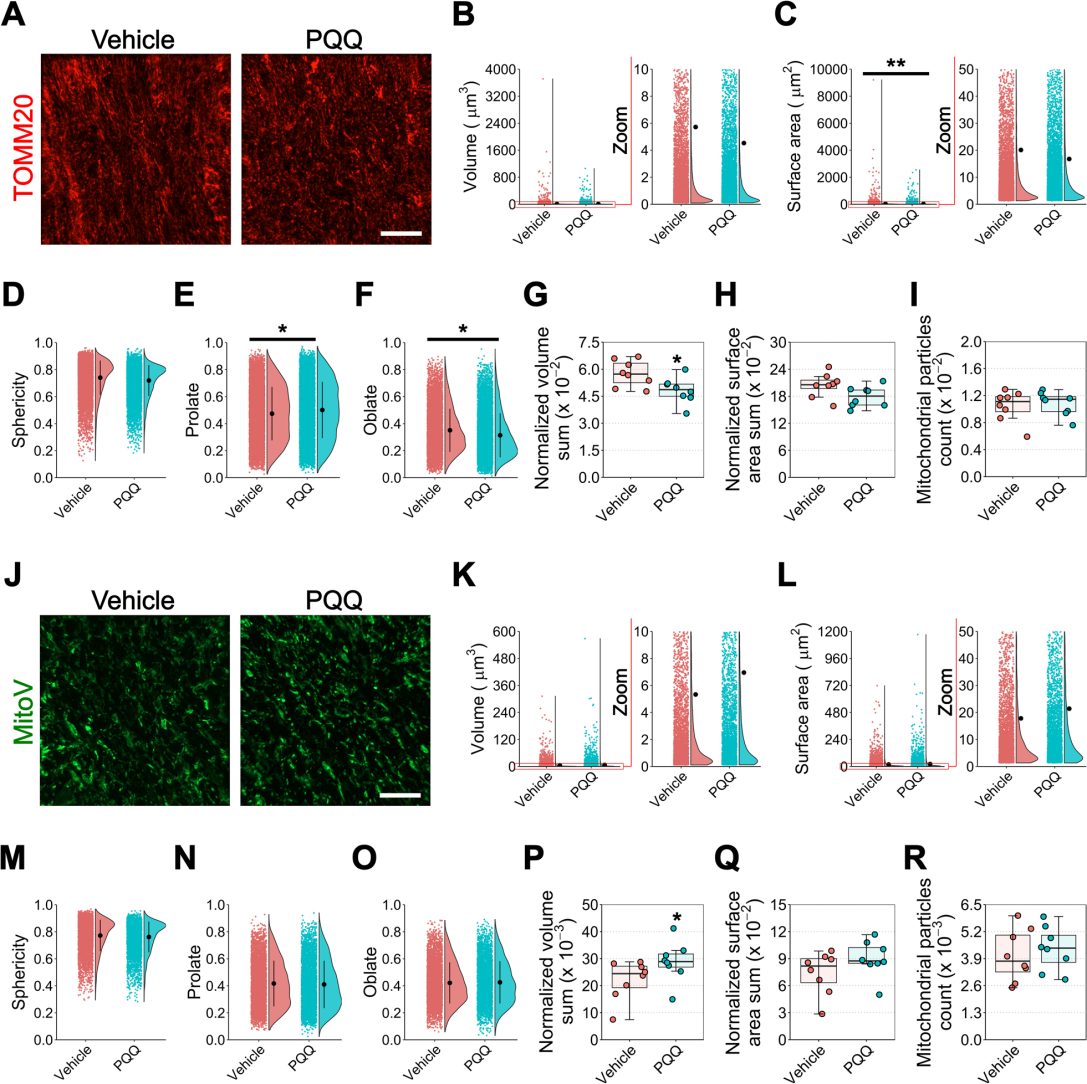
Effects of PQQ administration on general and RGC-specific mitochondrial morphology in the optic nerve in vivo. **A** Representative images of optic nerve longitudinal sections from MitoV animals treated long-term with either vehicle or 20 mg/kg PQQ immunolabeled for TOMM20 (red). **B**–**F** Violin plots showing individual volume (**B**), surface area (**C**), sphericity (**D**), prolate (**E**) and oblate (**F**) of TOMM20-positive mitochondrial particles in optic nerves from vehicle- or PQQ-treated animals. n = 9338 vehicle and 9293 PQQ disconnected particles in 8 different optic nerves per group. **G**–**I** Averaged volume sum (**G**), surface area sum (**H**) and count (**I**) per optic nerve of TOMM20-positive particles. n = 8 optic nerves per group. **J** Representative images of optic nerve longitudinal sections from MitoV animals treated long-term with either vehicle or 20 mg/kg PQQ immunolabeled for YFP (an antibody anti-GFP was used to limit loss of signal from potential bleaching of YFP; the staining is indicated here as MitoV) (green). **K**–**O** Violin plots representing individual volume (**K**), surface area (**L**), sphericity (**M**), prolate (**N**), oblate (**O**) of MitoV-positive particles in optic nerves of vehicle and PQQ-treated animals. n = 4378 vehicle and 3947 PQQ disconnected particles in 8 different optic nerves per group. **P**–**R** Averaged volume sum (**P**), surface area sum (**Q**) and count (**R**) per optic nerve of MitoV-positive particles. n = 8 optic nerves per group. For individual parameters, individual values of disconnected particles from each optic nerve were analyzed together and a linear mixed effects model was applied to account for the multiple observations that come from the same sample. Scale bar = 20 μm. The red zoom depicts the inset of data points to optimally visualize the data distribution. *p < 0.05 and **p < 0.01 versus vehicle

We next assessed whether mitochondrial morphological remodeling occurs following tissue mRNA and metabolic changes. We first assessed the effects of PQQ on gross mitochondrial morphology reconstructing TOMM20-positive mitochondrial particles in retinal GCL/NFL, IPL and in optic nerve. Mice were administered 20 mg/kg of PQQ via i.p. for 2 weeks and then euthanized and their tissue processed for high resolution confocal microscopy. No change in total mitochondrial morphology was observed following PQQ administration in the retina (Fig. 5; Additional file 8: Fig. 7) whereas optic nerves from PQQ-treated mice displayed a significant decrease in individual mitochondrial particle total surface area and changes to sphericity (Fig. 7A–I). A similar trend was identified at an average level per optic nerve (Additional file 10: Fig. 9A–E). As TOMM20 positive particles are representative of mitochondria from multiple cell types, we next determined whether there was an RGC-specific response in these tissues using our recently published mitochondrial reporter mouse which expresses YFP under a rat neuron-specific *Eno2* promoter and localized to mitochondria through a *Cox8a* gene-targeting signal fused to the YFP N-terminus. This strain is called MitoV (V for visual system) and the expression of YFP is specifically restricted to RGCs in the inner retina (with the expression in a subset of bipolar neurons and photoreceptors in the outer retina) [7]. A significant increase in individual sphericity was observed in the GCL of PQQ treated retinas, whereas no differences were detected in all the other parameters analyzed (Fig. 6). Taken as an average, all morphological parameters were similar between vehicle- and PQQ- treated mice (Additional file 9: Fig. 8). In the optic nerve, the administration of PQQ resulted in no changed individual particles morphology, although on average the mean volume and the volume sum were significantly increased (Fig. 7J–R; Additional file 10: Fig. 9F–J). Taken together, all the changes identified both at the molecular and morphological level may reflect some heterogeneity and variability across tissues and within conditions, suggesting that the overall effect of PQQ administration results in only a mild effect on mitochondrial content.

### PQQ modifies the metabolic profiles in non-diseased RGCs

**Fig. 8:**
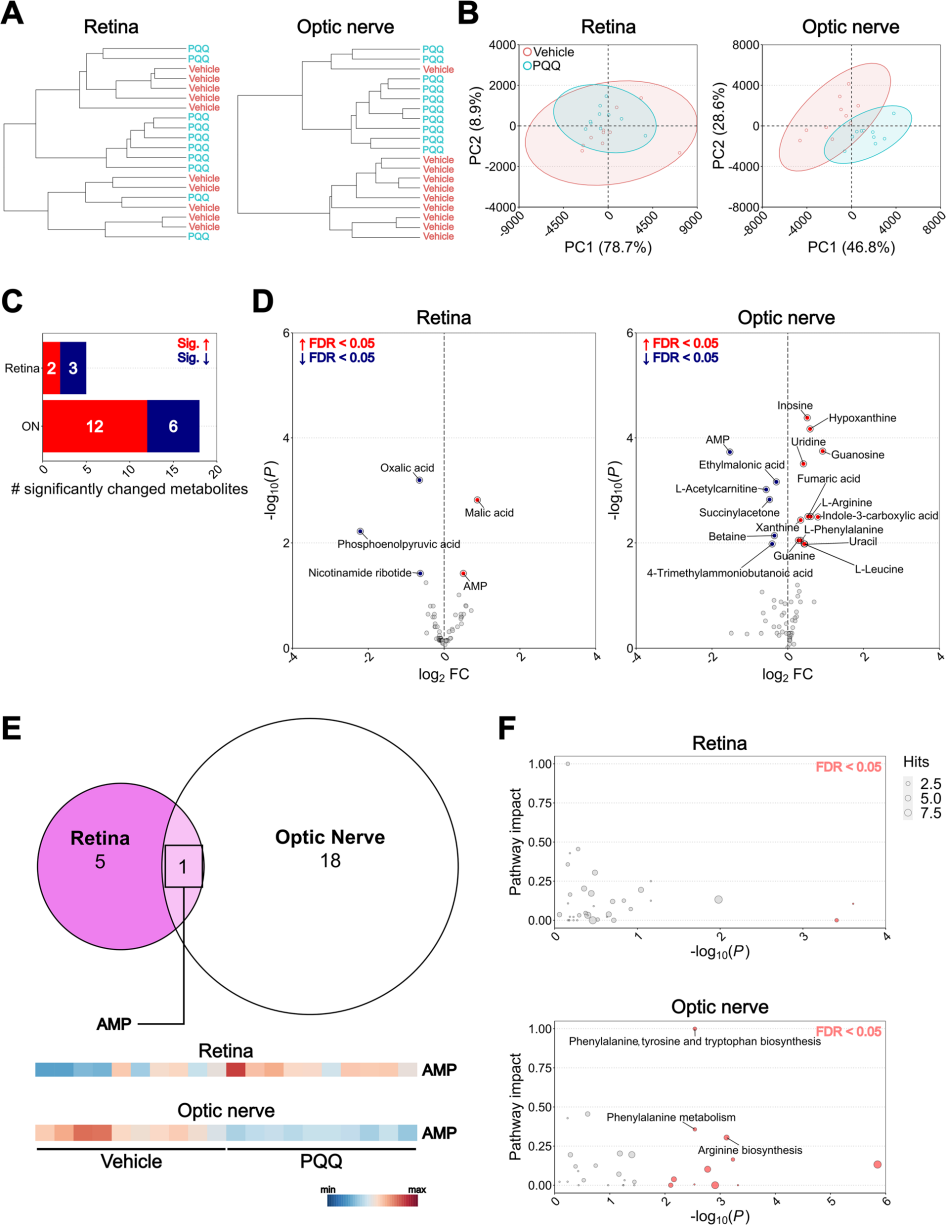
Effects of PQQ administration on metabolic profile of non-diseased RGC- related tissues in vivo. **A**, **B** Dendrograms (**A**) and principal component analysis (PCA; **B**) of retinal and optic nerve samples collected from mice treated with either vehicle or a single injection of 20 mg/kg PQQ after 24 h. **C**, **D** Bar chart (**C**) and volcano plots (**D**) indicating respectively the number and the increase/decrease of significantly changed metabolites in retinas and optic nerves from PQQ-treated animals compared to their vehicle-injected controls (FDR < 0.05; red = increased in PQQ, blue = decreased). **E** Euler plot and heatmaps showing commonly changed metabolites across tissues. The red and the blue in the heatmaps indicates respectively the highest and the lowest value by row. **F** KEGG pathway analysis indicating the predicted affected pathways based on the detected changed metabolites in retinas and optic nerves from PQQ-treated mice. Pathways were highlighted in red when FDR < 0.05 and annotated when the impact was high. The size of the points indicates the pathways hits, underlining the number of the metabolites detected within the pathway. n = 10 retinas or optic nerves per group

Because we identified a mild effect of PQQ on mitochondrial content and that the ATP levels may be regulated by a crosstalk of several metabolic processes, we set out to determine whether PQQ administration altered the metabolic profile of RGCs under basal conditions. To achieve this, we performed metabolomics across retinas and optic nerves collected from mice treated with a single injection of PQQ after 24 h from the injection using a low molecular weight enriched metabolomics protocol (identifying 63 low molecular weight metabolites in the retina and 73 enriched in the optic nerve with high accuracy and confidence; Additional file 1: Data 1). Hierarchical clustering (HC) of both conditions and individual samples identified some heterogeneity across and within conditions in retinal samples, overall suggesting similarities between retinas from vehicle- and PQQ- treated mice. However, the division between samples in optic nerve was largely distinguished (Fig. 8A; Additional file 11: Fig. 10). Principal component analysis (PCA) confirmed a complete overlap between groups in retina, whilst identifying a clear separation of samples in the optic nerve (Fig. 8B). This suggests that PQQ exerts differing effects on the two tissues studied. Administration of PQQ resulted in 5 changed metabolites in the retina (2 increased, 3 decreased) and 18 in the optic nerve (12 increased, 6 decreased) (Fig. 8C, D; Additional file 1: Data 1). Comparison of changed metabolites across tissues identified AMP as a commonly changed metabolite, although demonstrating an increase in the retina and a decrease in the optic nerve (Fig. 8E). Pathway analysis revealed that PQQ-induced metabolite modifications are predicted to have a minimal impact on retinal pathways, whilst significantly affecting phenylalanine metabolism, and arginine, phenylalanine, tyrosine, and tryptophan biosynthesis in the optic nerve (Fig. 8F; Additional file 1: Data 1).

Taken together, our data support the potential for PQQ as an adjuvant supplement for human retinal diseases. As our study provides strong data for injectable routes, but not for oral routes, further development is required if PQQ is to be properly administered at the target doses orally in humans.

## Discussion

RGCs require strict control of ATP content due to their high activity levels. This requires the maintenance of functional mitochondria to provide a continuous supply of ATP predominantly derived from OXPHOS in mitochondria [1, 36]. An imbalance in mitochondrial metabolism results in bioenergetic insufficiency, rendering RGCs susceptible to neurodegeneration. Altered metabolism and mitochondrial dysfunction occur early in the pathogenesis of many retinal diseases such as glaucoma, ADOA, and LHON [2–8]. Improving metabolic and mitochondrial function to increase ATP content has been effective in counteracting RGC damage and reducing the progression of neurodegeneration [7, 37], suggesting the potential of ATP boosters as therapeutic compounds against RGC-related neurodegenerative diseases.

Our data demonstrate that PQQ administration drives an increase in ATP content in vitro in cells isolated from both cortical neurons and RGC-relevant tissues, suggesting that PQQ can be rapidly utilized to enhance the concentration of ATP. Despite similar effects in vitro, in vivo administration of PQQ provided a differential effect on ATP generation in visual system tissues as compared with cortex likely depending on possible differences in drug uptake across blood-retinal barrier as compared with blood–brain barrier [38]. In this respect, while ATP content in the cortex remained unaltered, the ATP-boosting effect of PQQ was consistently obtained in visual system tissues, with a sustained elevation of ATP over 3 days after administration. The potential role of PQQ as a potent inducer of ATP synthesis with durable effects was identified over the whole RGC-trajectory through the visual system including retina containing RGC soma and dendrites, optic nerve containing RGC axons, and superior colliculus containing RGC terminals. Noteworthy, variation in ATP content may not undoubtedly attributed to changes in RGC energetic balance but rather be the result of the integration of neural, glial and vascular cell types in each of the visual system tissues, whose different composition might explain the variability in response and time identified across these tissues. The variability in ATP-boosting effect of PQQ was also demonstrated by differences in its influence on NAD pool, one of the primary factors influencing ATP generation rate of OXPHOS. In this respect, PQQ’s effect on ATP generation appeared unrelated with changes in NAD pool in retina and optic nerve, while a significant increment in NAD pool was evident in superior colliculus.

Promoting mitochondrial biogenesis is a key mechanism to bolster OXPHOS and ATP production by modulating the number of functional mitochondria. In previous studies on Hepa1-6, HepG2 and NIH/3T3 cell lines, PQQ has been reported to bolster ATP production by stimulating mitochondrial biogenesis through the activation of PGC-1α, a major transcriptional coactivator of several biogenesis-related factors, among which TFAM plays an important role in mitochondrial DNA replication and repair [18, 19, 39, 40]. However, PQQ effect appears to be cell and system dependent, with different outcomes depending on the cell line and the doses tested [18, 19, 40]. In this respect, our data demonstrated that either mitochondrial content or the expression of *Pgc-1α* and *Tfam* were not altered in visual system tissues following short- or long-term administration of PQQ, thus suggesting that the ATP-boosting effect of PQQ is likely independent of mitochondrial biogenesis mechanisms. On the other hand, the short-term ATP-boosting activity of PQQ was associated with a significant variation in mitochondrial membrane potential, apparently unrelated to a direct effect on OXPHOS complexes activity. The long-term administration of PQQ resulted in a mild but significant influence on transcription and translation of mitochondrial complex-related markers together with a subtle variation in gross mitochondrial morphology. Taken together, these results suggest that the ATP-boosting activity of PQQ only partially consists in a direct effect on mitochondrial content and function, at least in healthy conditions.

To identify alternative metabolic mechanisms involved in ATP-boosting effects of PQQ, we performed low molecular weight metabolomics in retina and optic nerve. We demonstrated a clear distinction between untreated and PQQ-treated optic nerve, suggesting a PQQ-mediated change in metabolic profile. Pathway analysis predicted impact on phenylalanine metabolism, and arginine, phenylalanine, tyrosine, and tryptophan biosynthesis. Changes in such pathways have also emerged in the metabolome profile of retina and optic nerve after the administration of nicotinamide, another relevant ATP-boosting compound providing strong neuroprotection in the retina via a metabolic mechanism [7]. Most of the metabolites displaying a significant increment following PQQ administration are strictly involved in anaplerotic mechanisms providing substrates to improve local ATP generation by fueling glycolysis or TCA cycle and OXPHOS [41]. In particular, the PQQ-dependent increase of L-arginine and fumarate can be derived from the metabolization of argininosuccinate in a reaction catalyzed by the argininosuccinate lyase [42]. Fumarate supports the activity of the TCA cycle and could contribute to the increased PQQ-mediated ATP content. Fumarate and its derivatives have also been demonstrated to exert antioxidant effects by regulating the activation of antioxidant pathways and resulting in enhanced cytoprotective cellular resistance to free radicals [43, 44]. Similarly, l-arginine may act as precursor of several endogenous polyamines reported to be neuroprotective in neurological and retinal diseases through the regulation of antioxidant, anti-inflammatory and anti-apoptotic mechanisms [45–49]. The antioxidant effects of both metabolites may further influence ATP production. In effect, end products of oxidative stress-induced lipid peroxidation have been reported to alter ATP synthase subunits resulting in impaired enzyme activity with reduced conversion of ADP to ATP and energy depletion [50, 51]. Reducing oxidative stress may therefore indirectly buffer the depletion of ATP under stress, suggesting an additional role of these metabolites in regulating the ATP pool but may not explain an increase in ATP seen in un-stressed systems. Low molecular weight metabolomics also revealed a PQQ-driven increment in l-leucine, a branched-chain amino acid which has multiple functions in the brain involving the metabolism of key neurotransmitters (glutamate), protein synthesis, and energy production [52]. l-leucine has been reported to induce ATP synthesis alone or in combination with other branched-chain amino acids by improving glycolysis and protecting RGCs from degeneration in a model of glaucoma, suggesting a potential metabolic substrate involved in the bioenergetic support of RGCs [53]. In addition to a, likely direct, effect in regulating ATP levels, the metabolism of l-leucine might provide carbon skeletons to increase energy substrates fueling TCA cycle, such as acetyl-CoA, and ketone bodies (acetoacetyl-CoA) synthesis [54]. Ketone bodies in turn may be used by astrocytes, neurons, and oligodendrocytes to further obtain acetyl-CoA under conditions of metabolic stress and glucose deprivation, resulting in improved metabolism, ATP production and RGC protection over stress [55, 56]. Supporting this, a ketogenic diet has been demonstrated to be neuroprotective in an experimental mouse model of glaucoma, with improved RGC survival, ameliorated axonal transport, and reduced gliosis [56]. Glial cells have the capacity to metabolize branched-chain amino acids, and neurons may use these metabolites either in vitro or in vivo under severe mitochondrial dysfunction to replenish TCA pool intermediates, suggesting a likely alternative way to promote metabolism and regulate the local ATP pool [55, 57, 58].

During RGC injury, intracellular ATP seems to initially increase as an early adaptation to sustain the high energy demand generated by the insult, declining gradually over time as the damage persists [59]. RGCs rapidly use ATP to sustain the high cellular activity and axonal transport of proteins required for remodeling and maintenance of cell cytoskeleton under stress, hence requiring a quick ATP turnover to constantly have the necessary metabolic substrate for their functioning [60]. Thus, if ATP synthesis is inadequate in maintaining constant optimal ATP levels under stress, RGC consume most of the ATP pool leading to metabolic exhaustion over time. For this reason, energy-boosting strategies providing RGCs more ATP might be neuroprotective in the context of disease. Supplementation of exogenous ATP in models of RGC injury gave different results under different type of stress. Supporting this, a single supplementation of ATP at either high or low dose in a model of optic nerve crush failed to protect RGCs from death [59]. However, multiple administration of ATP encapsulated in liposomes has been effective in protecting RGC from degeneration in a model of retinal ischemia/reperfusion [61]. The discrepancy in the outcomes might depend on the administration route, the duration of the injury, and the poor stability of ATP which might require multiple supplementations to become effective, raising the question if increasing ATP directly by its exogenous supplementation is an effective strategy to counteract RGC degeneration. Given its ability to increase local ATP content, PQQ might be a promising compound supplying RGCs with substrates counteracting stress-deriving bioenergetic insufficiency and the resultant neurodegeneration.

We tested the neuroprotective efficacy of PQQ in different models of RGC injury where bioenergetic capacity has been compromised. We initially assessed PQQ neuroprotection using an ex vivo model of retinal axotomy which results in Wallerian degeneration and loss of ATP with significant RGC degeneration 3 days post-axotomy [7, 34, 62, 63]. Our data demonstrated that the administration of PQQ confers RGC neuroprotection in this model, with an effective reduction of RGC cell loss and stress-related features such as nuclear shrinkage. The moderate PQQ neuroprotection reflects the complexity of factors regulating RGC degeneration in this context and might suggest that PQQ protection may act only on some of the neurodegenerative mechanisms in this model (e.g. supplementing ATP but not addressing neuroinflammation or caspase activity) [7, 34, 64]. Considering the ATP-boosting capacity of PQQ demonstrated in healthy conditions, a possible mechanism of PQQ neuroprotection may be ascribed, at least in part, to a likely counteraction of bioenergetic insufficiency through an increased ATP reservoir. To isolate and further investigate the potential contribution of PQQ in reducing RGC stress by regulating neuronal bioenergetic balance, we tested PQQ neuroprotection in a model where impaired bioenergetic capacity represents the principal insult driving RGC death. To test this we used an in vivo model of bioenergetic injury initiated by the inhibition of mitochondrial Complex I bioenergetic injury initiated by the inhibition of mitochondrial Complex I following intravitreal injection of rotenone [35, 65]. Rotenone injection induces acute mitochondrial damage, resulting in ATP depletion and oxidative stress which in turn cause RGC degeneration. This models a typical feature of RGC-specific retinal diseases such as glaucoma and genetic optic atrophies characterized by genetic mutations in mitochondrial-related genes as in the case of ADOA and LHON [2, 5, 6, 8]. The preservation of RGC-density and nuclear diameter resulting from PQQ administration supports the strong neuroprotective efficacy of PQQ. Since in this model RGC viability is strongly related to altered ATP reservoirs and PQQ has an ATP-boosting activity, the neuroprotective effects of PQQ are likely due to an amelioration of cell bioenergetic capacity by supporting ATP levels.

## Conclusions

Taken in concert, our data demonstrate that PQQ is neuroprotective in different models of RGC stress. PQQ administration increases local ATP content and alters metabolic profiles in non-diseased visual system tissues. The prominent neuroprotective efficacy of PQQ against RGC damage under a variety of stressors is possibly related to its ATP boosting activity, although a clear correlation would need further investigations. The present findings support a potential role of PQQ as a novel neuroprotective compound used as adjuvant with other current therapies to improve RGC resilience with a low risk of side effects. Although our data demonstrated a negligible effect of short-term oral delivery, the dietary supplementation with PQQ still remains a valid option given the possibility to improve its bioavailability by favoring the mobility across body barriers (e.g. absorption in the gastrointestinal tract and blood–brain barrier permeability) and by designing slow-release formulations. Further studies are needed to identify the optimal dose and delivery system in humans to achieve similar effects in promoting ATP content in health and disease before PQQ could be considered ready for clinical use.

### Supplementary Information

**Additional file 1: Dataset 1.** Raw mass spectrometry data (area under the curve), weight normalized results, comparisons, and pathway analysis.

**Additional file 2: Figure 1.** Assessment of PQQ interference on ATP assay in vitro. Luminescence of control samples with PQQ diluted in HBSS (control vehicle) at different concentrations (0.1, 0.5, 1, 5, 10, 50 µM) without cell lysates. n = 3 different replicates per group.

**Additional file 3: Figure 2.** Supplementary analysis of retinal cell survival in ex vivo and in vivo models of RGC stress. (A, B) Quantification of DAPI positive cell density per 0.01 mm^2^ (A) and mean DAPI nuclear diameter (B) in GCL of retinas cultured ex vivo. Retinal explants were cultured in either basic or supplemented media with either 50 or 100 μM PQQ for 3 days ex vivo (DEV). Control retinas (0 DEV) were directly fixed and processed after the dissection. n = 5 (0 DEV), 7 (3 DEV), 4 (3 DEV + 50 μM PQQ), 6 (3 DEV + 100 μM PQQ) retinas. (C, D) Quantification of DAPI positive cell density per 0.01 mm^2^ (C) and mean DAPI nuclear diameter (D) in GCL of retinas from animals injected either with DMSO (control) or rotenone and treated with vehicle or 20 mg/kg i.p. PQQ. n = 9 DMSO, 9 DMSO + PQQ, 10 rotenone and 9 rotenone + PQQ retinas. GCL, ganglion cell layer. *p < 0.05, **p < 0.01 and ***p < 0.001 versus 0 DEV (explants) or DMSO (rotenone model); ^#^*p* < 0.01 and ^##^*p* < 0.001 versus 3 DEV (explants) or rotenone (rotenone model).

**Additional file 4: Figure 3.** Effects of PQQ administration on cell viability in vitro. Evaluation of PQQ cell toxicity in dissociated mouse brain cortical cells incubated with 50 μM PQQ for 2 h. Cells maintained in HBSS for the same time were used as controls. Toxicity was assessed by Trypan blue assay and quantified as the number of cells/mL. n = 3 different cell suspensions from different hemispheres.

**Additional file 5: Figure 4.** Effects of PQQ administration by drinking water on ATP and NAD levels in visual system tissues in vivo. (A) ATP and (B) NAD content in retina, optic nerve, superior colliculus and brain cortex measured from mice treated with either vehicle or 20 mg/kg PQQ diluted in drinking water after 24 h. n = 6 animals per group. *p < 0.05 versus vehicle.

**Additional file 6: Figure 5.** Effects of PQQ administration on short term transcriptional activation of mitochondrial biogenesis in vivo. (A) mtRNA/nuRNA ratio in whole retinal samples from animals treated with a single i.p. injection of either vehicle or 20 mg/kg PQQ, calculated using the expression of *mt-Co2* and *Rsp18* as mitochondrial and nuclear reference gene, respectively. mtRNA/nuRNA ratio was measured 24, 48 or 72 h after the treatment. (B) *Pgc-1α* and *Tfam* mRNA levels measured in whole retinas from animals injected with either vehicle or 20 mg/kg PQQ after 24, 48 or 72 h. *Rsp18* was used as housekeeping gene. n = 6 vehicle and 7 PQQ retinas for 24 h, 8 vehicle and 7 PQQ retinas for 48 h, 8 vehicle and 7 PQQ retinas for 72 h.

**Additional file 7: Figure 6.** Full quantified and uncropped representative blots of Western Blot data. (A, B) Full blots of markers of mitochondrial complexes (ATP5a, UQCRC2, mt-CO1, SDHB, NDUFB8; blots on the left) in either retinas (A) or optic nerves (B) from vehicle-or PQQ-treated animals. β-actin was used as loading control after membrane stripping and reprobing (blots on the right). The optical density (OD) of each marker was normalized for the relative OD of the β-actin to provide the quantification reported in Fig. 3G, H. Since total OXPHOS rodent WB antibody cocktail (ab110413, Abcam) used to detect bands recognizes 5 markers contemporarily, two different exposures were performed to obtain the optimal visualization of bands (top = lower exposure; bottom = higher exposure). Black arrows indicate which marker was quantified on each membrane (top = ATP5a, UQCRC2 and SDHB; bottom = mt-CO1 and NDUFB8). Rat heart mitochondrial extract provided by the manufacturer (ab110341, Abcam) was diluted at 1:200 and run as positive control (PC). (C, D) Uncropped membranes of the representative blots shown in Fig. 3G, H.

**Additional file 8: Figure 7.** Supplementary analysis of retinal gross mitochondrial morphology after PQQ administration in vivo. Individual (violin plots) and averaged (box plots) analysis of reconstructed TOMM20-postive particles in GCL/NFL (A-G) and IPL (H-N) in retinas of mice after long term treatment either with vehicle or PQQ. For individual parameters, a linear mixed effects model was applied to account for the multiple observations that come from the same retina. Individual parameters in GCL/NFL (555 vehicle and 812 PQQ) or in IPL (1318 vehicle and 1361 PQQ) were measured on disconnected TOMM20-positive particles from 7 different retinas per group. n = 7 retinas per group in the averaged graphs. GCL, ganglion cell layer. IPL, inner plexiform layer. NFL, nerve fiber layer.

**Additional file 9: Figure 8.** Supplementary analysis of RGC-specific retinal mitochondrial morphology after PQQ administration in vivo. Individual (violin plots) and averaged (box plots) analysis of reconstructed MitoV-positive particles in GCL/NFL (A-G) and IPL (H-N) in retinas of MitoV mice after long term treatment either with vehicle or 20 mg/kg PQQ. For individual parameters, a linear mixed effects model was applied to account for the multiple observations that come from the same retina. Individual parameters in GCL/NFL (176 vehicle and 268 PQQ) or in IPL (600 vehicle and 552 PQQ) were measured on disconnected MitoV-positive particles from 7 different retinas per group. n = 7 retinas per group in the averaged graphs. GCL, ganglion cell layer. IPL, inner plexiform layer. NFL, nerve fiber layer.

**Additional file 10: Figure 9.** Supplementary analysis of general and RGC-specific mitochondrial morphology in optic nerve in vivo. Averaged morphological parameters per sample in TOMM20- (A-E) or MitoV-positive (F-J) particles in optic nerves from MitoV mice treated long term with either vehicle or 20 mg/kg PQQ. n = 8 optic nerves per group. *p < 0.05 and **p < 0.01 versus vehicle.

**Additional file 11: Figure 10.** Hierarchical clustering of retinal and optic nerve individual samples based on the metabolic profiles derived from the low molecular weight metabolomics in vivo. Correlation heatmaps representing the hierarchical clustering of individual retinal and optic nerve samples collected from animals treated with a single injection of either vehicle or 20 mg/kg PQQ after 24 h. Heatmaps were created using the Spearman rank correlation on Metaboanalyst 5.0 platform (red = highest correlation, blue = lowest correlation). n = 10 retinas or optic nerves per group.

## Data Availability

All data generated or analyzed during this study are included in this published article [and its supplementary information files].

## Abbreviations

ADOA: Autosomal dominant optic atrophy
ADP: Adenosine diphosphate
AMP: Adenosine monophosphate
ANOVA: Analysis of variance
ATP: Adenosine triphosphate
ATP5a1: ATP synthase F1 subunit alpha
BSA: Bovine serum albumin
CNS: Central nervous system
CS: Citrate synthase
Cyt C: Cytochrome C
DAPI: 4′,6-Diamidino-2-phenylindole
DB: Decylubiquinone
DCPIP: 2,6-Dichlorophenolindophenol
DEV: Days ex vivo
DMSO: Dimethyl sulfoxide
DTNB: 5,5′-Dithiobis (2-nitrobenzoic acid)
FDR: False discovery rate
GCL: Ganglion cell layer
GFP: Green fluorescent protein
HBSS: Hank’s balanced salt solution
HC: Hierarchical clustering
IPL: Inner plexiform layer
KEGG: Kyoto Encyclopedia of Genes and Genomes
LD50: Lethal dose 50
LHON: Leber hereditary optic neuropathy
mt-CO1: Mitochondrially Encoded Cytochrome C Oxidase I
NAD: Nicotinamide adenine dinucleotide
NADH: Nicotinamide adenine dinucleotide + hydrogen (reduced)
NDUFB8: NADH:Ubiquinone Oxidoreductase Subunit B8
NFL: Nerve fiber layer
OD: Optical density
OXPHOS: Oxidative phosphorylation
PBS: Phosphate-buffered saline
PCA: Principal component analysis
PFA: Paraformaldehyde
PGC-1α: Proliferator-activated receptor-gamma coactivator alpha
PQQ: Pyrroloquinoline quinone
RBPMS: RNA-binding protein with multiple splicing
RGC: Retinal ganglion cell
SDHB: Succinate dehydrogenase complex iron sulfur subunit B
TBS: Tris-buffered saline
TCA: Tricarboxylic acid
TFAM: Mitochondrial transcription factor A
TOMM20: Translocase of outer mitochondrial membrane 20
UQCRC2: Ubiquinol-cytochrome c reductase core protein 2
YFP: Yellow fluorescent protein
