# Supplementary figures and images for: Pyrroloquinoline quinone drives ATP synthesis in vitro and in vivo and provides retinal ganglion cell neuroprotection
Source: Acta Neuropathol Commun. 2023 Sep 8;11:146. doi: 10.1186/s40478-023-01642-6 (PMC10486004; doi:10.1186/s40478-023-01642-6)

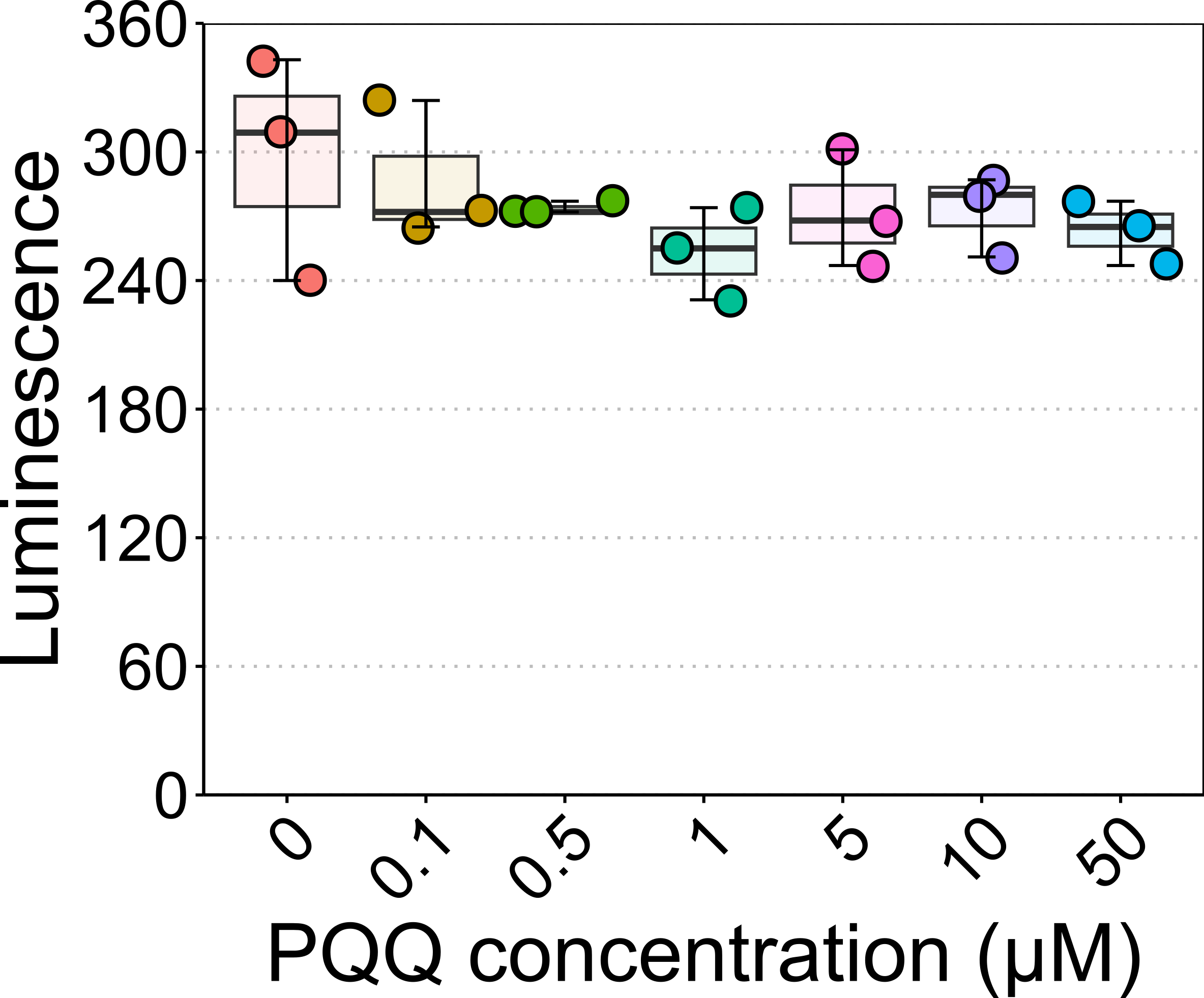

Supplement: Supplementary file 2 — Additional file 2: Figure 1. Assessment of PQQ interference on ATP assay in vitro. Luminescence of control samples with PQQ diluted in HBSS (control vehicle) at different concentrations (0.1, 0.5, 1, 5, 10, 50 µM) without cell lysates. n = 3 different replicates per group. [file 40478_2023_1642_MOESM2_ESM.tif]

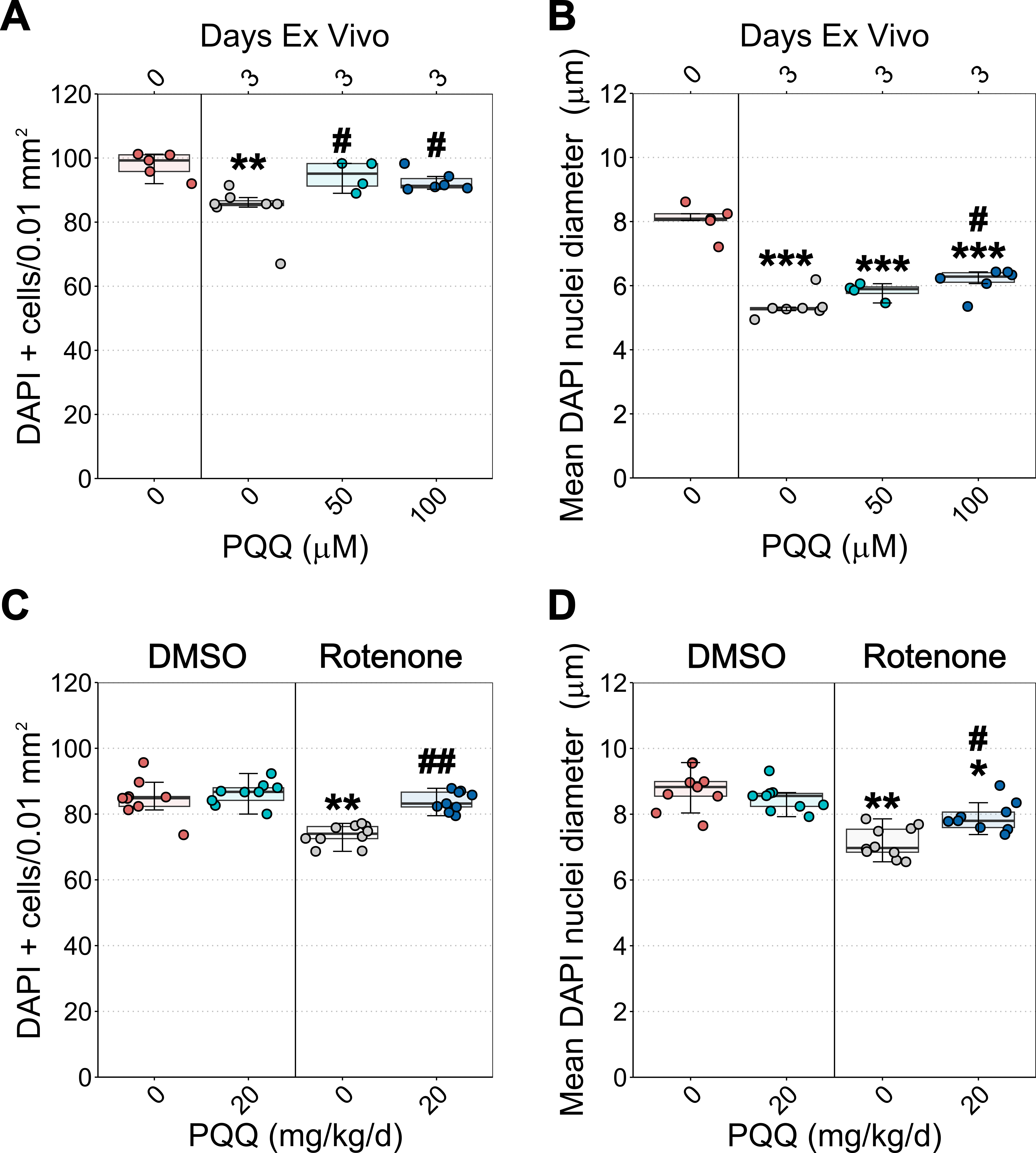

Supplement: Supplementary file 3 — Additional file 3: Figure 2. Supplementary analysis of retinal cell survival in ex vivo and in vivo models of RGC stress. (A, B) Quantification of DAPI positive cell density per 0.01 mm2 (A) and mean DAPI nuclear diameter (B) in GCL of retinas cultured ex vivo. Retinal explants were cultured in either basic or supplemented media with either 50 or 100 μM PQQ for 3 days ex vivo (DEV). Control retinas (0 DEV) were directly fixed and processed after the dissection. n = 5 (0 DEV), 7 (3 DEV), 4 (3 DEV + 50 μM PQQ), 6 (3 DEV + 100 μM PQQ) retinas. (C, D) Quantification of DAPI positive cell density per 0.01 mm2 (C) and mean DAPI nuclear diameter (D) in GCL of retinas from animals injected either with DMSO (control) or rotenone and treated with vehicle or 20 mg/kg i.p. PQQ. n = 9 DMSO, 9 DMSO + PQQ, 10 rotenone and 9 rotenone + PQQ retinas. GCL, ganglion cell layer. *p < 0.05, **p < 0.01 and ***p < 0.001 versus 0 DEV (explants) or DMSO (rotenone model); #p < 0.01 and ##p < 0.001 versus 3 DEV (explants) or rotenone (rotenone model). [file 40478_2023_1642_MOESM3_ESM.tif]

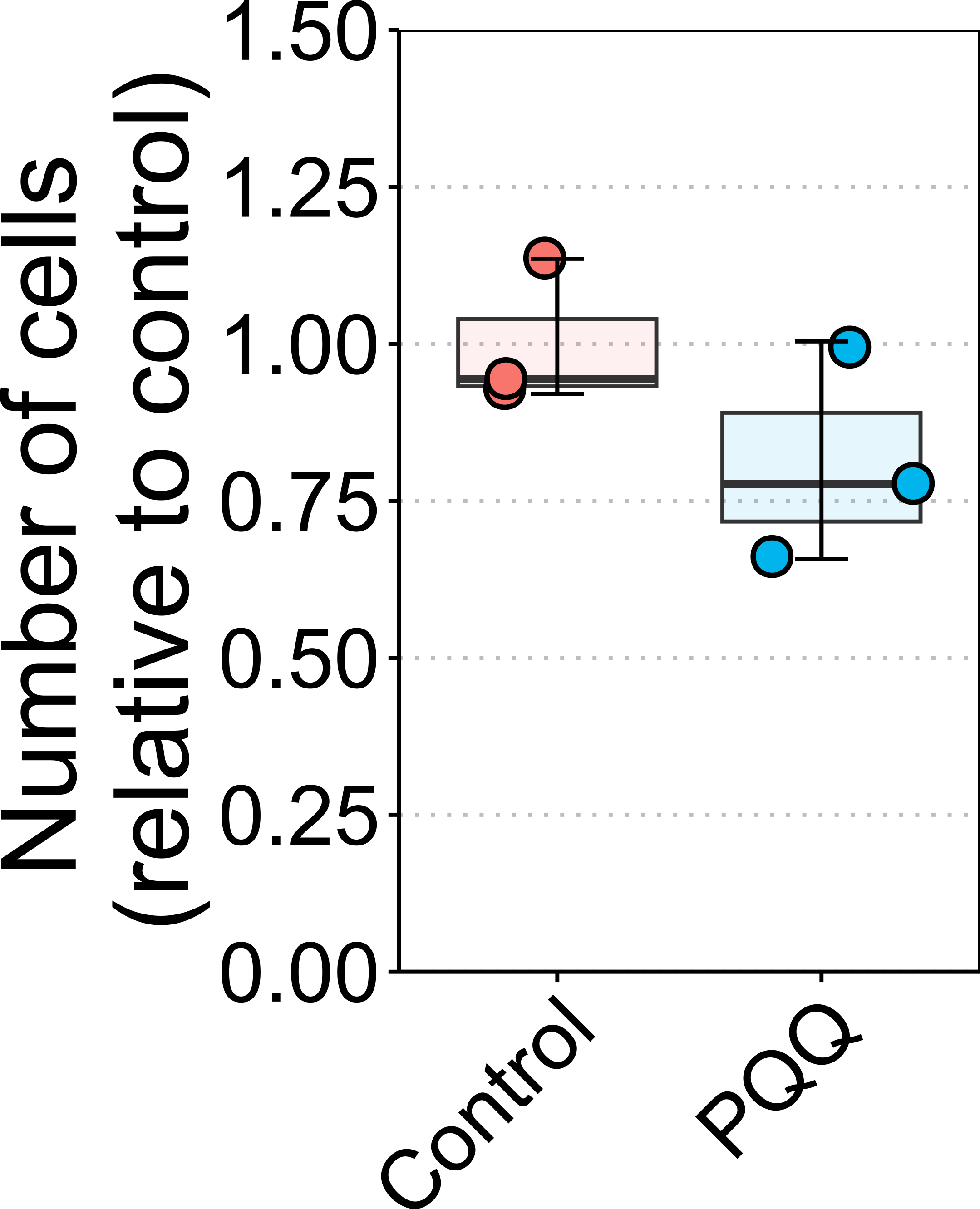

Supplement: Supplementary file 4 — Additional file 4: Figure 3. Effects of PQQ administration on cell viability in vitro. Evaluation of PQQ cell toxicity in dissociated mouse brain cortical cells incubated with 50 μM PQQ for 2 h. Cells maintained in HBSS for the same time were used as controls. Toxicity was assessed by Trypan blue assay and quantified as the number of cells/mL. n = 3 different cell suspensions from different hemispheres. [file 40478_2023_1642_MOESM4_ESM.tif]

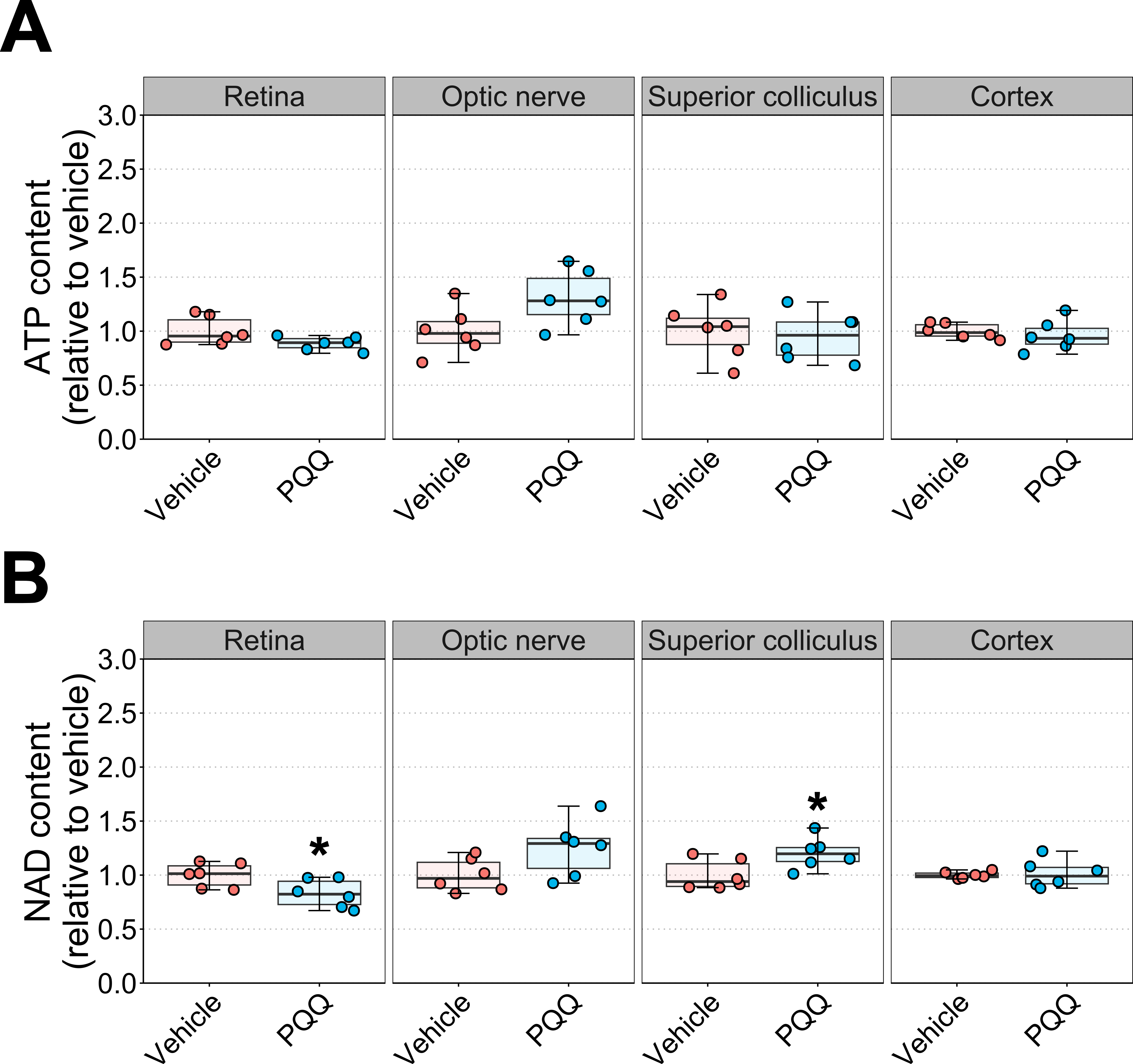

Supplement: Supplementary file 5 — Additional file 5: Figure 4. Effects of PQQ administration by drinking water on ATP and NAD levels in visual system tissues in vivo. (A) ATP and (B) NAD content in retina, optic nerve, superior colliculus and brain cortex measured from mice treated with either vehicle or 20 mg/kg PQQ diluted in drinking water after 24 h. n = 6 animals per group. *p < 0.05 versus vehicle. [file 40478_2023_1642_MOESM5_ESM.tif]

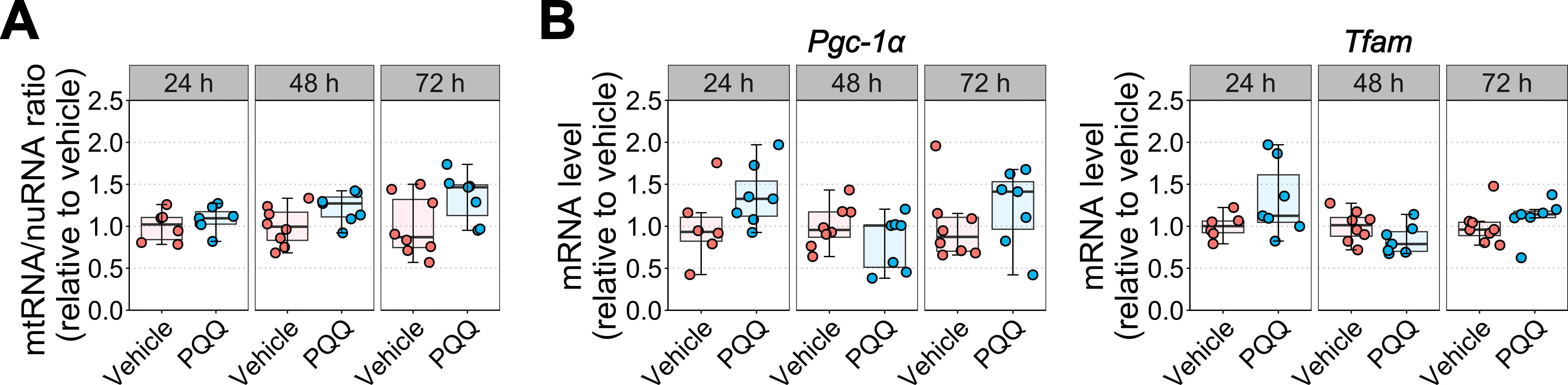

Supplement: Supplementary file 6 — Additional file 6: Figure 5. Effects of PQQ administration on short term transcriptional activation of mitochondrial biogenesis in vivo. (A) mtRNA/nuRNA ratio in whole retinal samples from animals treated with a single i.p. injection of either vehicle or 20 mg/kg PQQ, calculated using the expression of mt-Co2 and Rsp18 as mitochondrial and nuclear reference gene, respectively. mtRNA/nuRNA ratio was measured 24, 48 or 72 h after the treatment. (B) Pgc-1α and Tfam mRNA levels measured in whole retinas from animals injected with either vehicle or 20 mg/kg PQQ after 24, 48 or 72 h. Rsp18 was used as housekeeping gene. n = 6 vehicle and 7 PQQ retinas for 24 h, 8 vehicle and 7 PQQ retinas for 48 h, 8 vehicle and 7 PQQ retinas for 72 h. [file 40478_2023_1642_MOESM6_ESM.tif]

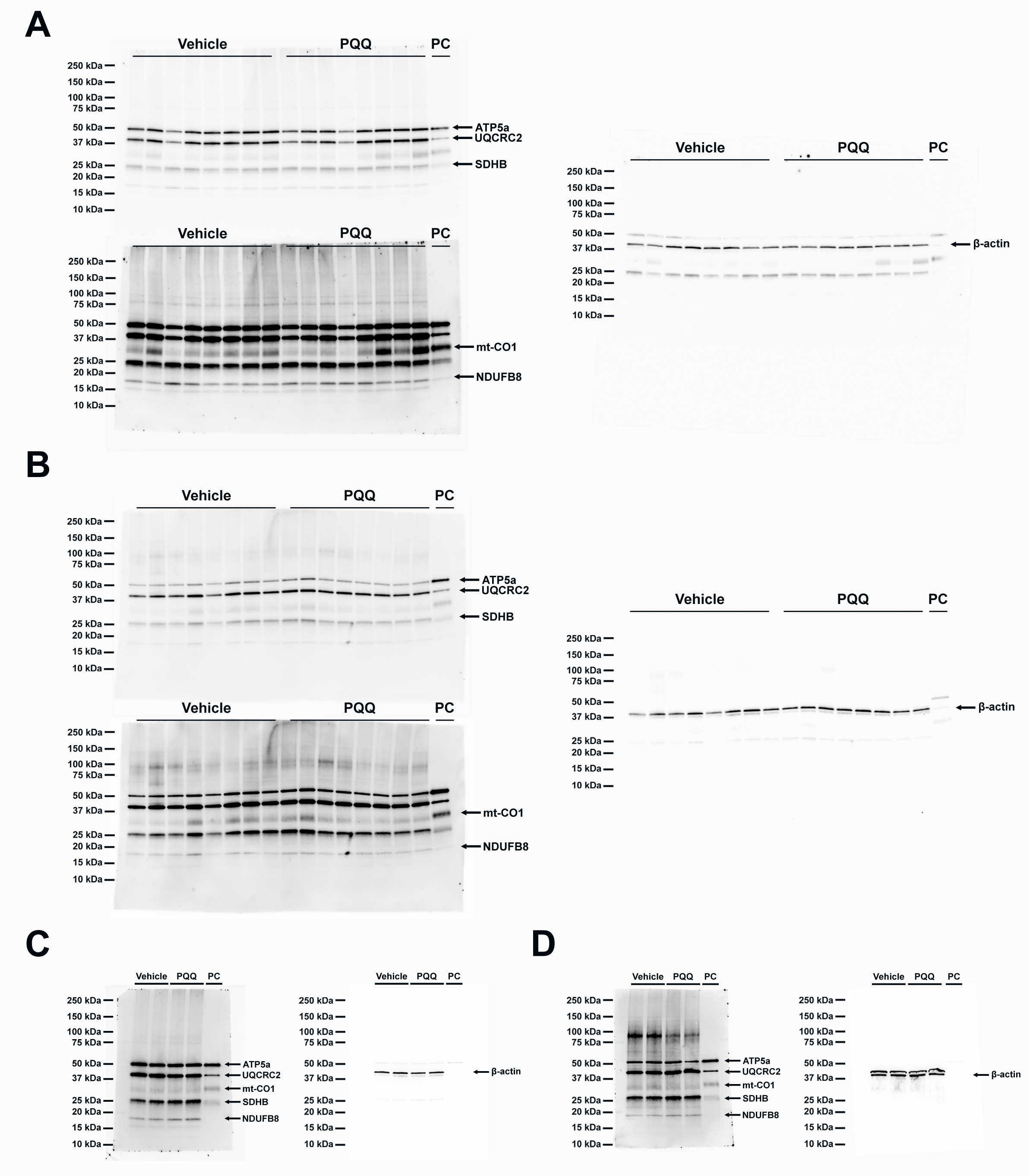

Supplement: Supplementary file 7 — Additional file 7: Figure 6. Full quantified and uncropped representative blots of Western Blot data. (A, B) Full blots of markers of mitochondrial complexes (ATP5a, UQCRC2, mt-CO1, SDHB, NDUFB8; blots on the left) in either retinas (A) or optic nerves (B) from vehicle-or PQQ-treated animals. β-actin was used as loading control after membrane stripping and reprobing (blots on the right). The optical density (OD) of each marker was normalized for the relative OD of the β-actin to provide the quantification reported in Fig. 3G, H. Since total OXPHOS rodent WB antibody cocktail (ab110413, Abcam) used to detect bands recognizes 5 markers contemporarily, two different exposures were performed to obtain the optimal visualization of bands (top = lower exposure; bottom = higher exposure). Black arrows indicate which marker was quantified on each membrane (top = ATP5a, UQCRC2 and SDHB; bottom = mt-CO1 and NDUFB8). Rat heart mitochondrial extract provided by the manufacturer (ab110341, Abcam) was diluted at 1:200 and run as positive control (PC). (C, D) Uncropped membranes of the representative blots shown in Fig. 3G, H. [file 40478_2023_1642_MOESM7_ESM.tif]

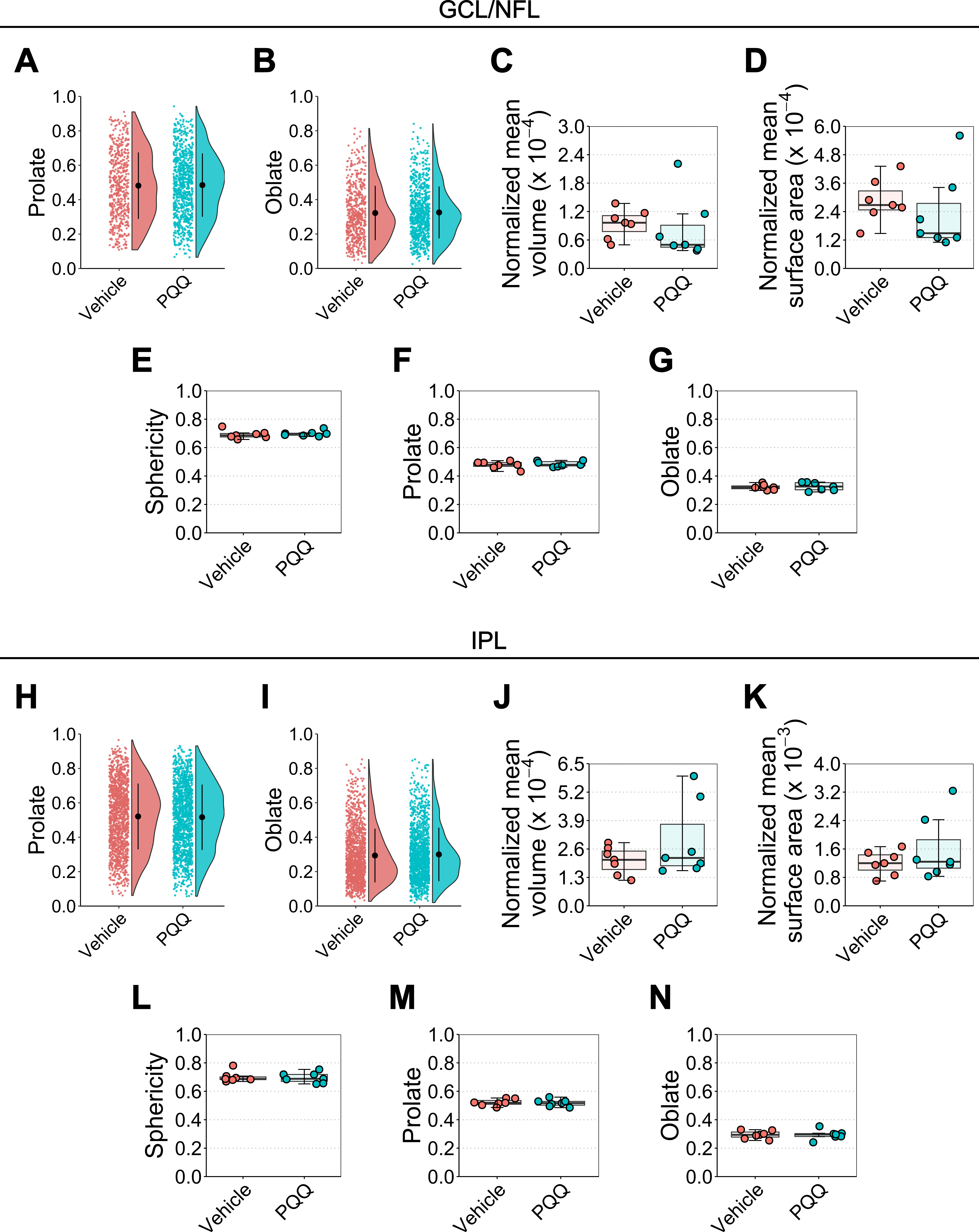

Supplement: Supplementary file 8 — Additional file 8: Figure 7. Supplementary analysis of retinal gross mitochondrial morphology after PQQ administration in vivo. Individual (violin plots) and averaged (box plots) analysis of reconstructed TOMM20-postive particles in GCL/NFL (A-G) and IPL (H-N) in retinas of mice after long term treatment either with vehicle or PQQ. For individual parameters, a linear mixed effects model was applied to account for the multiple observations that come from the same retina. Individual parameters in GCL/NFL (555 vehicle and 812 PQQ) or in IPL (1318 vehicle and 1361 PQQ) were measured on disconnected TOMM20-positive particles from 7 different retinas per group. n = 7 retinas per group in the averaged graphs. GCL, ganglion cell layer. IPL, inner plexiform layer. NFL, nerve fiber layer. [file 40478_2023_1642_MOESM8_ESM.tif]

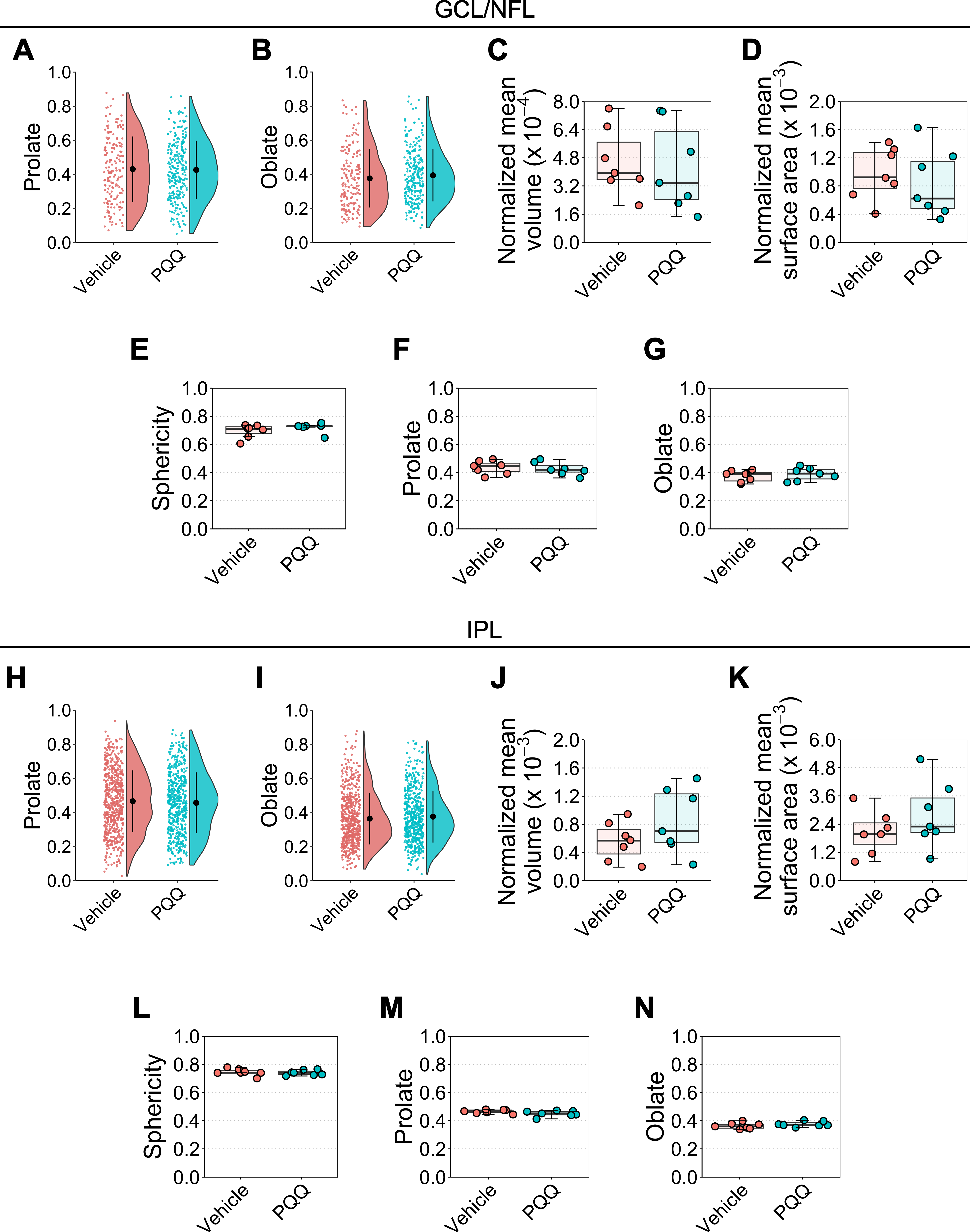

Supplement: Supplementary file 9 — Additional file 9: Figure 8. Supplementary analysis of RGC-specific retinal mitochondrial morphology after PQQ administration in vivo. Individual (violin plots) and averaged (box plots) analysis of reconstructed MitoV-positive particles in GCL/NFL (A-G) and IPL (H-N) in retinas of MitoV mice after long term treatment either with vehicle or 20 mg/kg PQQ. For individual parameters, a linear mixed effects model was applied to account for the multiple observations that come from the same retina. Individual parameters in GCL/NFL (176 vehicle and 268 PQQ) or in IPL (600 vehicle and 552 PQQ) were measured on disconnected MitoV-positive particles from 7 different retinas per group. n = 7 retinas per group in the averaged graphs. GCL, ganglion cell layer. IPL, inner plexiform layer. NFL, nerve fiber layer. [file 40478_2023_1642_MOESM9_ESM.tif]

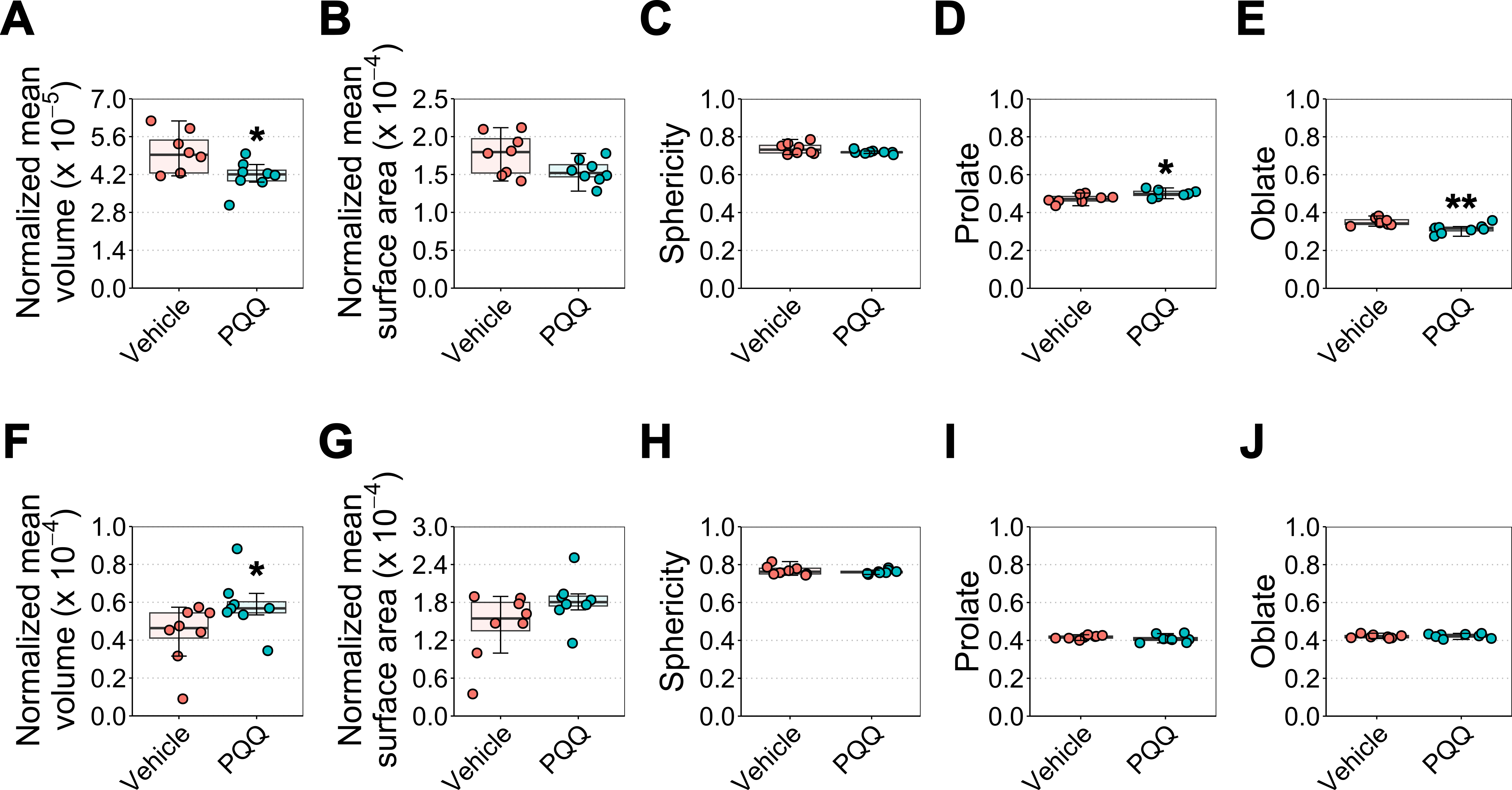

Supplement: Supplementary file 10 — Additional file 10: Figure 9. Supplementary analysis of general and RGC-specific mitochondrial morphology in optic nerve in vivo. Averaged morphological parameters per sample in TOMM20- (A-E) or MitoV-positive (F-J) particles in optic nerves from MitoV mice treated long term with either vehicle or 20 mg/kg PQQ. n = 8 optic nerves per group. *p < 0.05 and **p < 0.01 versus vehicle. [file 40478_2023_1642_MOESM10_ESM.tif]

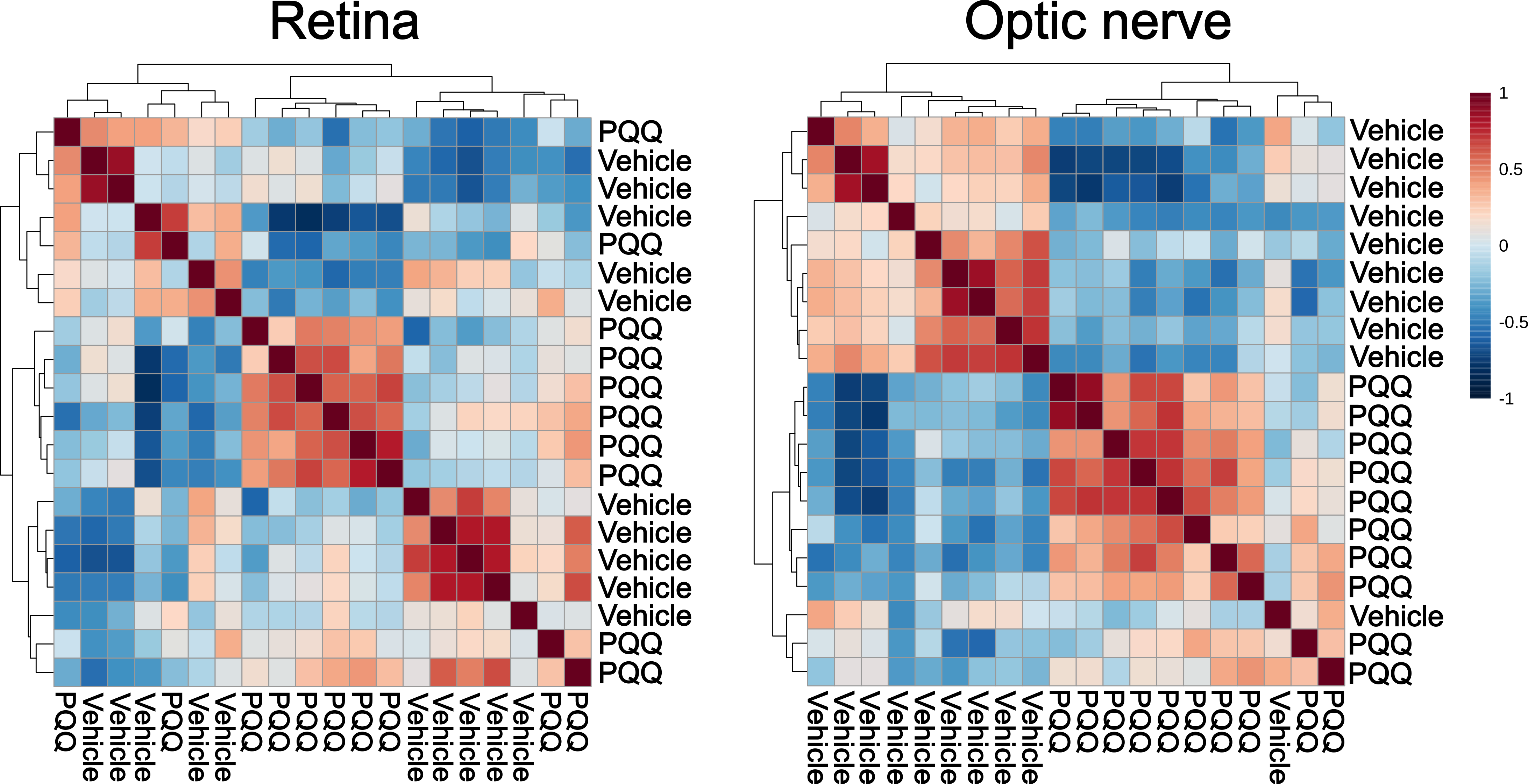

Supplement: Supplementary file 11 — Additional file 11: Figure 10. Hierarchical clustering of retinal and optic nerve individual samples based on the metabolic profiles derived from the low molecular weight metabolomics in vivo. Correlation heatmaps representing the hierarchical clustering of individual retinal and optic nerve samples collected from animals treated with a single injection of either vehicle or 20 mg/kg PQQ after 24 h. Heatmaps were created using the Spearman rank correlation on Metaboanalyst 5.0 platform (red = highest correlation, blue = lowest correlation). n = 10 retinas or optic nerves per group. [file 40478_2023_1642_MOESM11_ESM.tif]
